# Endohedral anionic nine-atom Zintl clusters of the elements tin and lead with lithium counterions

**DOI:** 10.1039/d5sc08011h

**Published:** 2026-01-09

**Authors:** C. E. Fajman, D. M. Dankert, P. Coburger, W. Klein, T. F. Fässler

**Affiliations:** a Technical University of Munich, TUM School of Natural Sciences, Chemistry Department, Chair of Inorganic Chemistry with Focus on New Materials Lichtenbergstraße 4 85748 Garching Germany thomas.faessler@lrz.tum.de; b Technical University of Munich, Catalysis Research Centre Ernst-Otto-Fischer-Straße 1 85748 Garching Germany

## Abstract

Intermetalloid clusters are considered as highly charged soluble models for intermetallic phases. While their bonding situation is not fully understood, their solubility makes them promising candidates for the fabrication of nanostructured intermetallic materials. We report the synthesis of endohedral [TM@Tt_9_]^4−^ (Tt = Sn and TM = Fe, Co, Ni, Pd; Tt = Pb and TM = Pd, Pt) clusters from solutions of K_4_Sn_9_ and the appropriate transition metal complex. The potassium ions are replaced with lithium cations through a simple salt metathesis reaction using LiCl. The intermetalloid cluster compounds adopt crystal structures, which are – except for the presence of the TM at the cluster center – isotypic either to the related Li/Sn_9_/en or Li/Pb_9_/en compounds containing unfilled clusters. The new compounds are further investigated by Raman, NMR, and EPR spectroscopy.

## Abstract 2

A synthetic protocol for generating a series of polyhedral cluster anions that are endohedrally filled with transition-metal atoms and paired with lithium counterions is reported.

## Graphical abstract

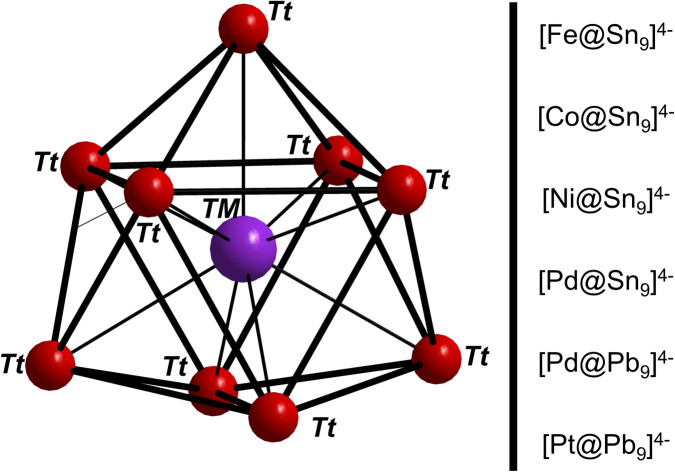

## Introduction

Over the last few decades, *Zintl* clusters have been extensively studied in solution and solid-state chemistry.^1–3^ In particular, ligand-free binary cluster anions containing transition metals surrounded by tetrel elements represent an increasingly diverse material class with complicated architectures. Structural resemblances in the atom arrangements between intermetalloid clusters and intermetallic compounds indicate that such clusters represent a unique model compound at the border of traditional molecular complexes and solid-state intermetallic compounds.^4,5^ Experimentally, a broad range of single- or multiple-atom endohedral group 14 clusters are known, ranging from 9 to 44 atom units [TM_*n*_@Tt_*m*_]^*x*−^.^1–3,6–10^ The smallest known intermetalloid clusters are nine-atomic clusters filled with a transition metal [TM@Tt_9_]^*x*−^, which can be either synthesised in solution from a combination of homoatomic deltahedral clusters with low-valent transition metals as the metal source,^11,12^ or prepared directly in the solid state,^13–15^ possibly followed by extraction of the preformed endohedral clusters from the solid state into solution.^16–18^ From solid state reactions only a small number of discrete endohedrally filled nine-atomic clusters such as in K_5_CoSn_9_,^14^ K_13_CoSn_17_,^15^ Na_12_Ni_1−*x*_Sn_17_,^13,15^ K_12_Pd_1−*x*_Sn_17_ (ref. 13) and K_4_RhPb_9_ (ref. 13) are known. In most reported endohedral clusters, the central transition metal (TM = Ru, Co, Ni or Cu) possesses a d^10^ electron configuration, while the charge of the known filled cluster ranges from −6 to −3. Endohedrally filled clusters that are obtained from reactions in solution might lead to larger clusters due to disproportionation reactions. For instance, [TM@Tt_10_]^*x*−^ anions are obtained by dissolving K_4_Tt_9_ in the presence of organometallic complexes. The resulting ten atomic clusters [TM@Tt_10_]^*x*−^ adopt three different structures such as a unique pentagonal prismatic coordination environment of the central Co and Fe atom in [Co@Ge_10_]^3−^,^19^ [Fe@Ge_10_]^3−^,^20^ a *C*_2v_-symmetric structure with two square faces as in [Fe@Sn_10_]^3−^,^21^ [Rh@Sn_10_]^3−^,^22^ or Ni atom centred bicapped square antiprismatic *closo-*[Ni@Pb_10_]^2−^.^23^ Even larger clusters have been obtained either as filled icosahedral units such as [Ru@Sn_12_]^4−^,^24^ [Rh@Sn_12_]^3−^,^22^ [Ir@Sn_12_]^3−^,^25^ [TM@Pb_12_]^2−^ (TM = Mn,^26^ Ni, Pd, Pt^23^) and [TM@Pb_12_]^3−^ (TM = Co, Rh, Ir,^27^ Au^28^) or fused endohedral clusters such as [TM_2_@Ge_17_]^4−^ (TM = Co, Ni^29^), [TM_2_@Sn_17_]^4−^ (TM = Co,^14,30^ Ni,^31^ Rh,^22^ Pt^32^), [Pd_2_@Tt_18_]^4−^ (Tt = Ge,^33^ Sn^34^), and [Ni_3_@Ge_18_]^4−^.^35^ More impressive examples of multiple fused clusters such as [Rh_3_@Sn_24_]^5−^,^22^ [Cu_4_@Tt_18_]^4−^ (Tt = Sn, Pb),^36^ [Au_8_Pb_33_]^6−^,^37^ [Au_12_Pb_44_]^8−^,^37^ and [Au_3_Ge_45_]^9−^^38^ show the potential for step by step cluster growth to large intermetalloid aggregates on the way to intermetallic phases.^39,40^ However, the mechanism of cluster growth is still under debate and not fully understood. The most probable path seems to be a stepwise synthesis, in which a transition metal complex first coordinates to the cluster, followed by removal of the ligand and subsequent formation of larger intermetalloid units.^40,41^ Indication for this can be found for example in [Au_3_Ge_45_]^9−^, the intact [Ge_9_]^4−^ cluster skeleton is linked by covalent Ge–Ge exo bonds, which are formed by oxidation of two adjacent cluster units. Nevertheless, the reaction pathways towards those structures are not fully understood and most likely some more intricate intermediate reaction steps may take place. For a better understanding of the reaction mechanisms the fundamental knowledge of the bonding situation over a broad range of transition metals in the basic building units [TM@Tt_9_]^4−^ must first be established.^42,43^

Over the last few years, attempts to obtain tetrel element clusters filled with transition metal atoms *via* typical reactions from ethylenediamine have resulted in the majority of cases in the formation of larger fused clusters, but only a few with filled nine-atom variants.^22,31,32^ Recently, we have shown that tetrel element polyhedral [Tt_9_]^4−^*Zintl* clusters can be obtained by dissolution of K_4_Tt_4_ phases in the presence of LiCl, and two main features have been observed after these reactions. On one hand, exchange of the potassium ions for lithium ions in ethylenediamine as a result of a simple salt metathesis reaction takes place. On the other hand, we observed partial oxidation of the clusters, leading to the formation of nine-atom clusters from four-atom clusters as well as to the formation of cluster dimers [Ge_9_–Ge_9_]^6−^ from [Ge_9_]^4−^ clusters.^44^ According to reported formations of larger fused transition metal main group element clusters^31,34,45^ we elaborated a synthetic protocol to generate a series of clusters filled endohedrally with a zero-valent transition metal atom in the presence of lithium ions.

## Results and discussion

### Synthesis and crystal structure data

Based on our previous experiments involving LiCl,^44^ the new compounds [Li_4_(en)_8_][Fe_0.059(3)_@Sn_9_] (1), (K[18]crown-6)_2_K_2_[Fe_0.055(4)_@Sn_9_]·1.5 en (2), and [Li_4_(en)_8_][Co_0.827(5)_@Sn_9_] (3) are obtained by reacting K_4_Sn_9_ with (IPr)TM(DVTMS) (IPr = 1,3-bis-(2,6-diisopropylphenyl)-1,3-dihydro-2*H*-imidazol-2-ylidene, TM = Fe or Co, and DVTMS = divinyltetramethyldisiloxane) in ethylenediamine (en). The reaction is carried out in the presence of four equivalents of LiCl for compounds 1 and 3, or in the presence of two equivalents of 18-crown-6 for compound 2. After filtration of the en solution, the filtrate is layered with toluene to promote crystallization. Compounds [Li_4_(en)_8_][Ni@Sn_9_] (4), [Li_4_(en)_8_][Pd_0.824(4)_@Sn_9_] (5), [Li_4_(en)_8_][Pd_0.460(5)_@Pb_9_] (6) and [Li_4_(en)_8_][Pt_0.426(4)_@Pb_9_] (7) were synthesized under reaction conditions analogous to those used for compounds 1 and 3. The key variation of the synthesis protocol lies in the choice of zero-valent transition metal sources, with Ni(COD)_2_ (COD = cyclooctadiene) and TM(PPh_3_)_4_ (TM = Pd, Pt) as the zero-valent sources of Ni, Pd and Pt, respectively.

Single crystals isolated from the reactions revealed that all compounds contain at least partially endohedrally filled nine-atom clusters. The amounts of the respective transition metal atoms differ considerably within the tetrel element cluster. In compounds 1 and 2, the Fe sites within the [Sn_9_]^4−^ clusters are occupied by about 6%. Unfortunately, a refinement of the Fe atom position with anisotropic displacement parameters was not possible for compound 1 due to the low scattering power of the crystals. Because all attempts to obtain better single crystals with Li^+^ counterions failed, the crystallization was repeated with 18-crown-6 and resulted in 2. Here, the occupancy of the Fe atom position is 5.5% and, thus, close to the value of 1. The anisotropic refinement was possible in this case. The presence of iron in the single crystals was further confirmed by qualitative EDX measurements of single crystals (SI). While the cluster in compound 4 is fully occupied by Ni, the Co atom in compound 3 and the Pd atom in compound 5 exhibit occupancies of approximately 80%. In contrast, the central Pd and Pt atoms of the [Pb_9_]^4−^ clusters in compounds 6 and 7 are occupied by less than 50%, respectively. For all presented compounds, we determined the charge of the respective cluster anion from the presence of four Li^+^ or K^+^ ions per cluster entity, and therefore, the charge of the cluster is assigned to −4. Accordingly, no change in the overall charge of the filled cluster in comparison to the corresponding unfilled cluster is observed. The appearance of [TM@*E*_9_]^4−^ clusters is obvious for the uncharged group 10 elements Ni, Pd, and Pt,^12,13,15^ which have a d^10^ electron configuration. Assuming the presence of d^10^ electron configurations, [Cu@Sn_9_]^3−^ and [Cu@Pb_9_]^3−^ are achievable from en solutions.^11^ Higher charged endohedral clusters such as [Co@Ge_9_]^5−^ as well as [Ru@Sn_9_]^6−^ are obtained from liquid ammonia solutions, and [Co@Sn_9_]^5−^ was observed in solvent-free solids.^14–16^ Notably, in all cases the charge correlates with a formal d^10^ electron configuration of TM. Cluster anions containing Fe in a d^10^ configuration would require a higher charge but are generally found in larger cluster frameworks from en solutions.^7,8,20^ This can be interpreted as a lower stability of higher charged Fe- or Co-atom filled nine-atom clusters. Thus, partially oxidized [Fe@Sn_9_]^4−^ and [Co@Sn_9_]^4−^ anions occur only in low concentrations in the solution, and by co-crystallizing with unfilled host [Sn_9_]^4−^ clusters an averaged low occupation of the clusters – as a mixture of [TM@Sn_9_]^4−^ and [Sn_9_]^4−^ – appears in the crystal structure. In all present Li salts (1 and 3 to 7) the anionic clusters are surrounded by a matrix of Li^+^ cations coordinated by four en molecules (SI Fig. S18–S24). Similar to the unfilled structures, the solvent cation structures form complex 3D coordination polymers.

**Fig. 1:**
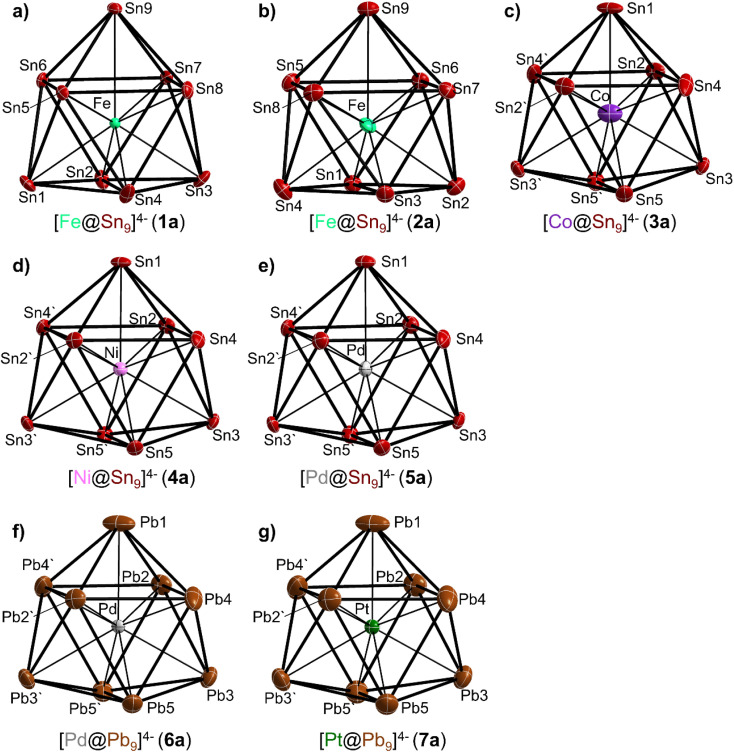
The endohedral clusters (a) 1a to (g) 7a. Displacement ellipsoids are set at the 50% probability level. All ellipsoids are shown anisotropic except for Fe in anion 1a, which is shown isotropic. Detailed bond lengths can be found in the SI.

Compounds 1 and 2 comprise the clusters [Fe_0.059(3)_@Sn_9_]^4−^ (1a) and [Fe_0.055(4)_@Sn_9_]^4−^ (2a), respectively (Fig. 1a and b). These two compounds adopt the same structure type as the unfilled variants [Li(en)_2_]_4_[Sn_9_]^32^ and [K_2_(18-crown-6)_2_K_2_]Sn_9_·1.5 en, respectively.^46^ The unit cell volumes of 1 and 2 are 2280.27(13) Å^3^ and 5422.2(2) Å^3^, respectively. Noticeably, the unfilled structure related to compound 1 shows an almost identical unit cell volume of 2279.46(17) Å^3^, while the unfilled structure related to compound 2 exhibits a slightly lower unit cell volume of 5380.2 Å^3^. Compounds 3 to 7, containing the filled anions 3a to 7a (Fig. 1c–g), all crystallize in the same structure type with space group *Pbcn*, analogous to [Li(en)_2_]_4_[Pb_9_], which contains the unfilled lead cluster.^44^ For compounds 3–7, the unit cell volumes are 4669.9(2) Å^3^, 4694.3(2) Å^3^, 4716.3(4) Å^3^, 4745.4(4) Å^3^ and 4722.35(19) Å^3^, respectively. In comparison, the unfilled variant has a slightly larger unit cell volume of 4699.6(3) Å^3^ than compounds 3 and 4, but a slightly lower unit cell volume than compounds 5–7. In this case, two opposing effects need to be considered. One is the incorporation of an atom into the central cavity of the [Tt_9_]^4−^ cluster, resulting in an increase in unit cell volume. The other effect arises from different cluster framework atoms, Sn for 3–5 as well as Pb, with a larger atomic radius, for 6 and 7. Therefore, a direct comparison of the unit cell volumes is difficult. The discussion of the cluster volumes provides a more suitable approach to analyse changes due to the insertion of an additional atom and will be addressed later.

Although the crystallographic point symmetry of the cluster anions in 1a and 2a is *C*_1_, their shapes are best described as slightly distorted *C*_4v_ symmetric monocapped tetragonal antiprisms, similar to the unfilled [Sn_9_]^4−^ clusters in [Li(en)_2_]_4_[Sn_9_]^32^ and [K_2_(18-crown-6)_2_K_2_]Sn_9_·1.5 en.^46^ In anions 1a and 2a there are three very different sets of Fe–Sn distances. The distance to the capping atom of the [Sn_9_]^4−^ unit is the longest, 2.841(8) Å and 2.812(13) for 1a and 2a, respectively. The distances to the capped square are the shortest, 2.416(8) to 2.476(12) Å and 2.450(13) to 2.478(13), and those to the open square face are slightly longer, 2.551(9) to 2.626(12) Å and 2.564(13) to 2.635(13) for 1a and 2a, respectively. A comparison with the distances from the centroid of [Sn_9_]^4−^ to the capping atom (2.7757(3) Å in 1a) indicates that the Fe atom is slightly shifted from the center of the quadratic antiprism towards the open square face. The clusters 3a to 7a have point symmetry *C*_2_, but appear as almost *D*_3h_ symmetric tricapped trigonal prisms also the same as the unfilled [Pb_9_]^4−^ anions in [Li(en)_2_]_4_[Pb_9_].^44^ Generally, in this structure type the cluster is generated by a twofold axis from an asymmetric unit containing one half of a cluster, so the high symmetry of the cluster originates from the space group symmetry and has to be considered with particular care. For instance, for unfilled [Sn_9_]^4−^ a monocapped square antiprism with *C*_4v_ symmetry is often found, and the investigation of [Co@Sn_9_]^4−^ and [Ni@Sn_9_]^4−^ solvates with K^+^ counterions shows *C*_4v_ symmetry in the solid state, too.^12,17^ Therefore, we cannot exclude that the observed cluster shape close to *D*_3h_ symmetry merely results from a superposition of different orientations of a *C*_4v_ symmetric cluster, possibly with an almost square planar face formed by Sn1/Sn2/Sn3/Sn4 as well as by Sn1/Sn2′/Sn3′/Sn4′ (and the corresponding Pb atoms in 6a and 7a). Supporting this, for all anions found in this structure type so far, distinctly elongated ellipsoids are observed for atoms Sn1/Pb1 and Sn4/Pb4 (see Fig. 1). However, the filled [Cu@Sn_9_]^3−^ and [Cu@Pb_9_]^3−^ clusters, crystallizing with K counterions in a different structure type, adopt *D*_3h_ symmetry, too.^11^ While the symmetry of the cluster does not necessarily change due to the presence of a transition metal atom at the cluster center, as shown by [Li(en)_2_]_4_[Pb_9_] and several compounds with filled clusters crystallizing in the same structure type, the size of the cluster anion sometimes triggers a different packing, which might be accompanied by a change in the number of co-crystallized solvent molecules or in the space group. This was observed, for example, within a series of three cluster [Ge_9_]^4−^, [Sn_9_]^4−^ and [Pb_9_]^4−^ anions, where the compound containing the largest one, [Pb_9_]^4−^, was found to crystallize in a structure type which differs from the Ge and Sn analogues.

For the anions 3a to 7a only two sets of different bond lengths are found, shorter distances between the central atom and the cluster atoms forming the trigonal prism, and longer distances to the capping atoms. As a result of this the [TM@Tt_9_]^4−^ polyhedra possess an almost spherical shape, and the contacts between transition metal atom and prism Tt atoms are in the narrow range of 2.5723(6) to 2.6747(8) Å for Sn and 2.6594(17) to 2.7153(11) Å for Pb, with slightly longer contacts to the capping atoms of 2.6264(13) to 2.7191(9) Å and 2.741(2) to 2.8172(12) Å, respectively. The Tt–Tt bond lengths of anions 1a to 7a are in the range of 2.9289(7) to 3.2880(5) Å and 3.1088(10) to 3.2339(10) Å for Sn–Sn and Pb–Pb, respectively, and are thus in the typical region for deltahedral Sn and Pb clusters.

The insertion of the transition metal atom into the centre of the cluster is accompanied by a significant increase in cluster volume (see Table 1). The cluster volumes of anions 1a and 2a are 33.2 Å^3^ each, corresponding to an increase in cluster size by about 0.6% compared to [Sn_9_]^4−^ with a cluster volume of 33.0 Å^3^. Anions 3a–5a exhibit a volume of 36.0 Å^3^, 36.6 Å^3^, and 38.4 Å^3^, reflecting volume increases by 9.1%, 10.9% and 17.3% relative to an unfilled [Sn_9_]^4−^ cluster, respectively. For the lead-based anions 6a and 7a, a cluster volume of 40.6 Å^3^ is observed in both cases, which corresponds to an increase in cluster size by about 5.7% compared to an unfilled [Pb_9_]^4−^ (38.7 Å^3^).

**Table 1:** Structural parameters such as prism heights *h*_1–3_ of an almost tri-capped trigonal prism (*D*_3h_) and ratios of diagonals *d*_1_ and *d*_2_ of the open planar square of a slightly distorted mono-capped square antiprism (*C*_4v_) for the endohedral clusters 1a to 7a, and as a reference the corresponding values of their unfilled equivalents in [Li(en)_2_]_4_[Sn_9_] and [Li(en)_2_]_4_[Pb_9_] are given

	*h*_1_	*h*_2_	*h*_3_	*d*_1/_ *d*_2_	Point group^a^	Volume [Å^3^]	Volume increase [%]	Occupation [%]
[Sn_9_]^4−^	1.00	1.01	1.24	1.08	*C*_4v_	33.0	—	—
[Fe_0.059(3)_@Sn_9_]^4−^ (1a)	1.00	1.02	1.25	1.08	*C*_4v_	33.2	0.5	5.9(3)
[Fe_0.055(4)_@Sn_9_]^4−^ (2a)	1.00	1.03	1.34	1.03	*C*_4v_	33.2	0.6	5.5(4)
[Co_0.827(5)_@Sn_9_]^4−^ (3a)	1.00	1.05	1.05	1.30	*D*_3h_	36.0	9.1	82.7(5)
[Ni@Sn_9_]^4−^ (4a)	1.00	1.02	1.02	1.24	*D*_3h_	36.6	10.9	100
[Pd_0.824(4)_@Sn_9_]^4−^ (5a)	1.00	1.02	1.02	1.20	*D*_3h_	38.4	17.3	82.4(4)
[Pb_9_]^4−^	1.00	1.09	1.09	1.39	*D*_3h_	38.7	—	—
[Pd_0.460(5)_@Pb_9_]^4−^ (6a)	1.00	1.06	1.06	1.32	*D*_3h_	40.6	5.7	46.0(5)
[Pt_0.426(4)_@Pb_9_]^4−^ (7a)	1.00	1.06	1.06	1.32	*D*_3h_	40.6	5.8	42.6(4)

a The nearly equal prism heights indicate an approximate *D*_3h_ symmetry for clusters 3a–7a,. However, crystallographically, these clusters possess only *C*_2_ point symmetry and are generated by a twofold axis from an asymmetric unit containing one half of the cluster. Thus, the apparent higher symmetry arises from space-group symmetry and must be interpreted with caution.

As pointed out earlier,^47^ the increase in cluster volume of (partially) filled clusters in comparison to unfilled [Sn_9_]^4−^ and [Pb_9_]^4−^ clusters is indicative for cluster filling.^16^ The small increase in the volume of iron centered anions 1a and 2a is due to the low occupancy of the central iron position of about 6%. Despite the similar size of Co and Ni, a larger volume increase of the cluster cage is observed for 4a in comparison to 3a because of a 17% higher occupied transition metal position. The highest volume change is found for 5a due to a high occupancy of 82% and the large atomic radius of Pd representing the only 4d element in this series of filled [Sn_9_]^4−^ clusters. A much smaller expansion of the cluster framework is observed for 6a because a much lower occupancy of 46% of the central Pd atom in 6a is found and the unfilled cluster [Pb_9_]^4−^ is already larger than the unfilled tin cluster. The latter fact means that lead clusters have more free space to accommodate transition metal atoms, and their bonds are not as strongly elongated than their tin analogues. Previous findings on [Cu@Tt_9_]^3−^ showed a similar trend (Tt = Sn, Pb). However, the comparison of [Cu@Sn_9_]^3−^ to 4a (both have a fully occupied central transition metal position) shows an anomaly because the expectedly smaller Cu^+^ ion (volume increase: 12.9%) should influence the cluster expansion less than the larger zero-valent Ni(0) atom (volume increase: 10.9%). In this comparison, mostly size effects are accounted for and no electronic interactions. Stronger back bonding from the cluster surface atoms to the central Cu^+^ ion than to the neutral Ni atom is expected. The interactions between Cu^+^ ions and anionic clusters can best be described as polarization or back bonding of the cluster electrons towards the central atom. This is also reflected in the natural charge density of +0.2 at the central Cu atom reported by Fässler *et al.*, which indicates some back bonding of the cluster towards the copper ion.^11^ Most literature known endohedral clusters show varying occupancies for the central atom, therefore we suggest to “normalize” the cluster expansion by dividing it by the occupancy of the transition metal atom position in order to allow a more appropriate comparison and discussion of the values. Since the degree of metal incorporation can depend strongly on the specific synthetic conditions, such as the choice of precursor, or reaction time and temperature, this normalization ensures that differences in cluster expansion reflect intrinsic structural trends rather than variations in occupancy.

**Fig. 2:**
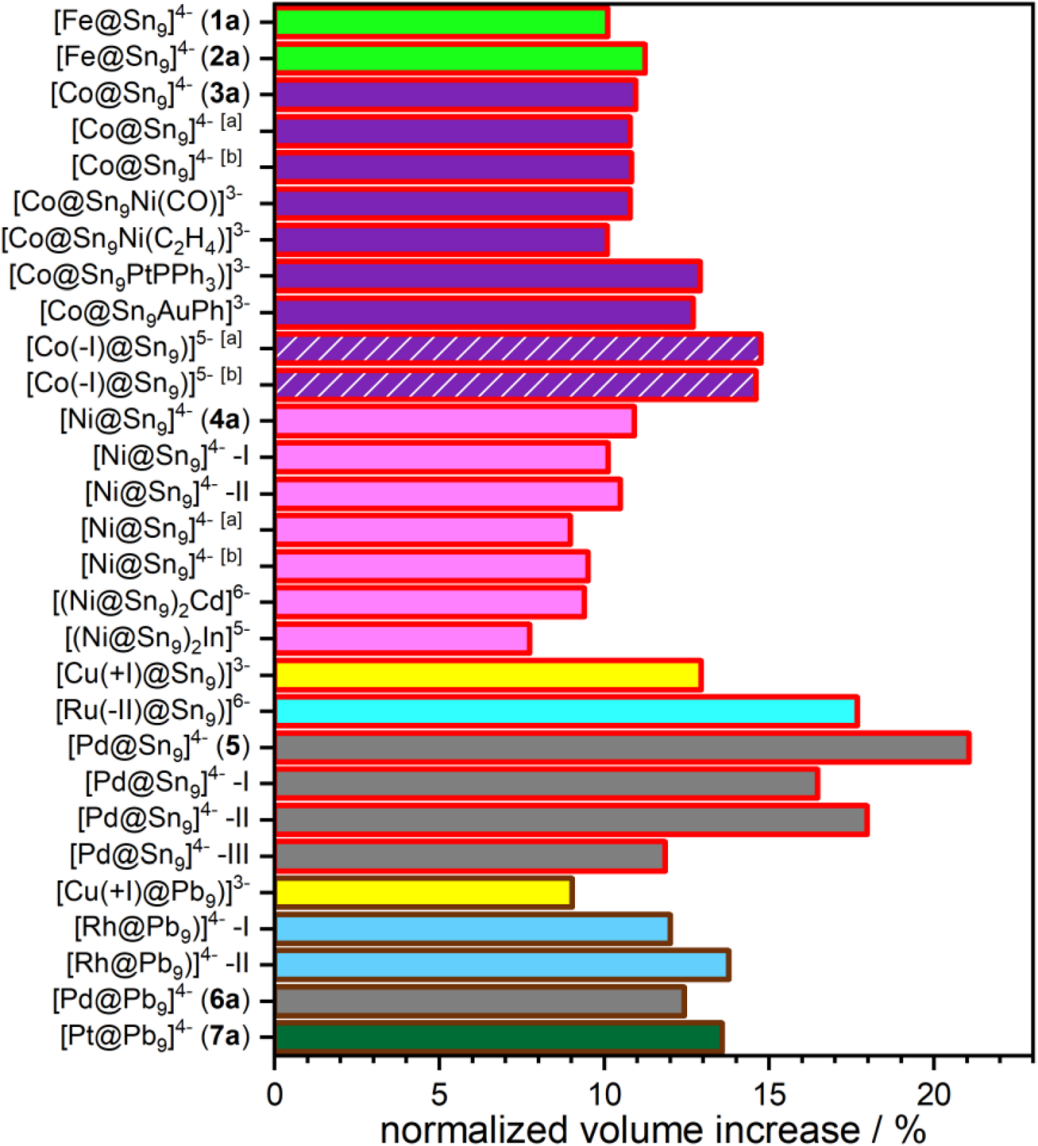
Normalized volume increase of endohedral clusters in anions 1a to 7a and of literature known endohedral clusters compared to their unfilled counterparts in a bar chart. The given normalized volume change (Δ*V/x*) is calculated by dividing the cluster expansion (Δ*V*) by the occupancy (*x*) of the TM atom position in the formula [TM_*x*_@Sn_9_]^4−^. The inner colors of the bars represent the central TM atoms while the colors of the bar frames indicate the cluster surface atoms. Fe, Co, Ni, Cu, Ru, Pd, Rh, Pt, Sn and Pb are shown as bright green, purple, pink, yellow, bright blue, grey, blue, dark green, red and brown, respectively. For clarity, the bars for [Co(−i)Sn_9_]^5−^ are dashed with white lines. If a cluster is present in more than one structure, it is indicated by [a] and [b]. In compounds with two crystallographically independent cluster anions the anions are individually analyzed and displayed with roman numbers. Reference data were taken from ref. 11, 12, 14–17, 44 and 48.

After the normalization of the volume increase, the Fe, Ni and Co filled clusters show comparable volume changes (Fig. 2), ranging between 10.1 and 11.1%. Also, the endohedral clusters, which are decorated with further organometallic fragments show a similar cluster expansion of 10.1% and 10.7% for [Co@Sn_9_Ni(L)]^3−^ (L = CO or C_2_H_4_)^17^ and of 9.4% for the dimeric [(Ni@Sn_9_)_2_Cd]^6−^.^48^ However, a clearly larger volume expansion (12.7% and 12.9%) for the Co clusters decorated with AuPh and PtPPh_3_ hints at a different binding situation. This is in good agreement with the reported quantum chemical calculation, which shows an interaction between the two metals and an increased electron density on the Co sites.^17^ Furthermore, earlier reports on [Ru(−ii)@Sn_9_]^6−^ reveal a much larger volume expansion of 18% for an endohedral nine-vertex tin cluster occupied by a Ru(−ii) anion. This massive volume increase is mostly related to the size of the formally negatively charged central atom.^16^

### Raman spectroscopy

**Fig. 3:**
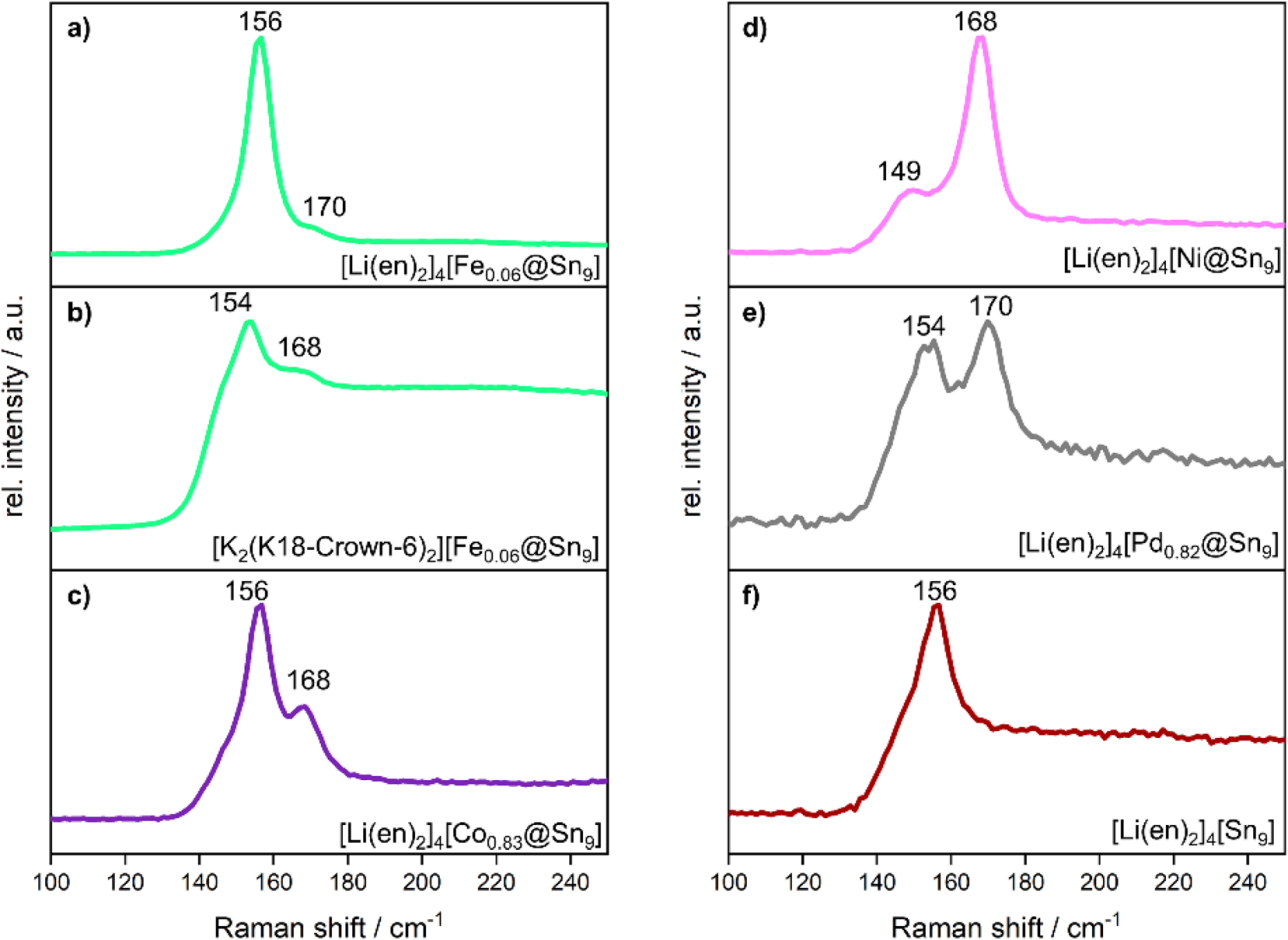
Raman spectra measured on single crystals of (a–e) compounds 1 to 5 and of (f) the unfilled cluster as a reference. The spectra in the range 50 – 800 cm^−1^ are shown in the SI.

Raman spectra for unfilled and endohedrally filled clusters for neat solids and clusters obtained from solution have been reported before. Thus, Raman spectroscopy opens up the possibility to derive information about vibrational behavior of products containing endohedral clusters.^13,16^ The Raman spectra of 1 to 5 reveal two distinct bands in the range of 154–156 cm^−1^ and 168–170 cm^−1^ (Fig. 3). Raman spectroscopy could not be performed for compounds 6 and 7 due to their sensitivity and rapid decomposition under irradiation with the laser. In compound 4, a weak band at 149 cm^−1^ is observed instead of the band in the range of 154–156 cm^−1^. For all compounds except 4, unfilled and filled clusters are overlapping in the structure. Thus, the observed bands in the range of 154–156 cm^−1^ correspond to the breathing modes of unfilled [Sn_9_]^4−^ clusters that have been reported at 156 cm^−1^.^44^ The remaining bands in the range of 168 and 170 cm^−1^ match the previously reported endohedral clusters [Pd@Sn_9_]^4−^ and [Ru@Sn_9_]^6−^ (at 171 cm^−1^).^13,16^ The appearance of these bands therefore serves as evidence for the presence of the filled and unfilled clusters. Notably, the position of these bands appears to be largely independent of the cationic framework, and the different central transition metal atoms show only a weak influence on the vibration around 170 cm^−1^. In compound 4, where the Ni site is fully occupied, the Raman spectrum lacks the band associated with unfilled clusters at 156 cm^−1^, instead, a weaker band at 149 cm^−1^ is observed, corresponding to the filled cluster. In compounds 1–3 and 5, this band is typically obscured by the broader and more intense signal of the unfilled cluster at 156 cm^−1^, occasionally appearing only as a weak shoulder.

This spectral assignment is further supported by previous reports on the vibrational behavior of [Sn_9_]^4−^ cluster anions, whose Raman spectra show characteristic and very intense breathing modes between 146 and 156 cm^−1^ for [Sn_9_]^4−^.^44^ Computational studies predict three characteristic bands for endohedral clusters such as [Pd@Sn_9_]^4−^ and [Ru@Sn_9_]^6−^ at 148 cm^−1^, 171 cm^−1^ and 260 cm^−1^, although in experimental spectra, typically only the most intense signal at 171 cm^−1^ is clearly discernible.^13,16^ In previously reported endohedral clusters, this shift of the breathing mode to higher wavenumbers (171 cm^−1^) compared to the unfilled cluster has been consistently observed.^13,16^

### NMR spectroscopy

For compounds 4 and 5^119^Sn NMR spectroscopy experiments were performed on single crystals dissolved in ethylenediamine and on the reaction solutions. For compound 7 only small quantities of single crystals were obtained, therefore the reaction solution was analyzed by ^207^Pb NMR spectroscopy (Table 2). Compounds 1-3 and 6 could not be investigated by NMR spectroscopy due to the paramagnetic nature of the former three compounds. In addition, we found rapid decomposition of these compounds in solution.

**Table 2:** Chemical shifts, coupling constants of compounds 4, 5 and 7 as well as relevant reported chemical shifts for various compounds in (a) ethylenediamine, (b) acetonitrile-d3, and (c) dimethylformamide. The abbreviation *m* denotes the atomic mass 63, 119, 195, and 207 of the isotopes of Tt = Sn and Pb) as well as TM = Cu and Pt respectively

Compound	*δ* ^*m*^Tt (ppm)	^117^Sn–^119^Sn (Hz)	^*m*^Tt–^*m*^TM (Hz)
[Ni@Sn_9_]^4−^ (4a)^a^	−1232	—	—
[Pd_0.824(4)_@Sn_9_]^4−^ (5a)^a^	−763, −1239	—, 283	—
[Sn_9_]^4−^ (ref. 44)^a^	−1239	283	—
[Ni_2_@Sn_17_]^4−^ (ref. 31)^a^	−1176	—	—
[Pd_2_@Sn_18_]^4−^ (ref. 45)^a^	−751	—	—
[Ni@Sn_9_H]^3−^ (ref. 49)^a^	−837	59	—
[Pd@Sn_9_H]^3−^ (ref. 50)^a^	−326	∼40	—
[Cu@Sn_9_]^3−^ (ref. 11)^b^	−1440	85	280
[Pt_0.426(4)_@Pb_9_]^4−^ (7a)^a^	−3062	—	4123
[Pb_9_]^4−^ (ref. 44)^a^	−4192	—	—
[Pt@Pb_12_]^2−^ (ref. 51)^c^	1780	—	3440

**Fig. 4:**
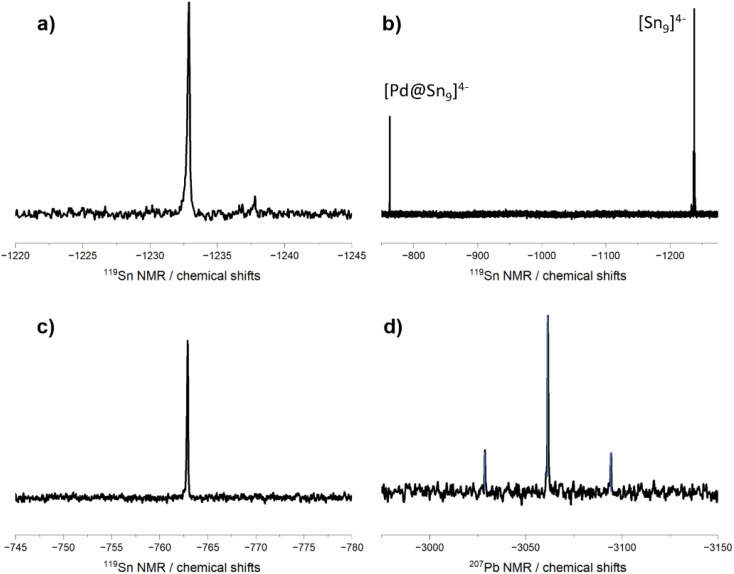
^119^Sn NMR spectra of compounds 4 (a) and 5 (b). In (c) an enlarged area shows the signal of the [Pd@Sn_9_]^4−^ cluster. The ^207^Pb NMR spectrum of anion 7a (d). All spectra were recorded in en.

The anions in compounds 4, and 5 as well as 7 exhibit characteristic ^119^Sn and ^207^Pb NMR resonances, respectively. In the ^119^Sn NMR spectra, chemical shifts are observed at −1232 ppm for compound 4 and at −763 ppm and −1239 ppm for compound 5. In the ^207^Pb NMR spectra, a signal at −3062 ppm is found (Fig. 4). For comparison the reported chemical shifts of [Sn_9_]^4−^ and [Pb_9_]^4−^ cluster anions with Li counterions are observed at −1239 ppm and −4192 ppm, respectively.^44^ Compound 4 only contains filled [Ni@Sn_9_]^4−^ clusters. The absence of other signals in the ^119^NMR spectra matches the exclusive presence of only filled clusters in compound 4. For compound 5 two signals at −1239 ppm and −763 ppm are observed. The multiplet at −1239 ppm, with a coupling constant of 283 Hz, is attributed to the unfilled [Sn_9_]^4−^ cluster. Consequently, the second signal at −763 ppm is assigned to the filled [Pd@Sn_9_]^4−^ cluster. The simultaneous observation of both signals in compound 5, which contains only partial Pd occupancy and thus both filled and unfilled clusters, indicates that there is no exchange of the central transition metal atom between clusters on the NMR timescale. While the chemical shifts for the signal of unfilled [Sn_9_]^4−^ and [Ni@Sn_9_]^4−^ anions are similar, the shift for the [Pd@Sn_9_]^4−^ anion is notably downfield shifted relative to the unfilled cluster. This pronounced deshielding is consistent with previously reported data for related Ni- and Pd-filled clusters, [Ni_2_@Sn_17_]^4−^ and [Pd_2_@Sn_18_]^4−^, which show signals at −1176 ppm and at −751 ppm, respectively.^31,45^

Although the nine-atom cluster [Cu@Sn_9_]^3−^ is structurally more comparable to anions 4a and 5a and would thus be expected to provide a meaningful NMR comparison, the respective measurements were conducted in different solvents (en for 4a and 5a and acetonitrile for [Cu@Sn_9_]^3−^). Consequently, a direct comparison of the chemical shifts is not pursued further due to potential solvent-induced effects. Furthermore, no inter-skeletal ^117^Sn–^119^Sn coupling and no coupling to the central transition metal atoms are found in anions 4a and 5a. This absence can be attributed to the quadrupolar nature and low natural abundance of the relevant transition metal isotopes (Ni and Pd), as well as the resulting fast quadrupolar relaxation, which leads to broadening or complete loss of both TM-Sn and Sn–Sn scalar couplings in the NMR spectrum.^31,45^ These observations are consistent with the findings in the fused endohedral clusters [Pd_2_@Sn_18_]^4−^ (ref. 45) or [Ni_2_@Sn_17_]^4−^.^31^ For the analogous protonated [TM@Sn_9_H]^3−^ cluster skeletal ^117^Sn–^119^Sn coupling constants of 59 and 43 Hz have been observed (for TM = Ni or Pd), therefore the coupling in our comparable compounds is arguably too small to be resolved.^49,52^ In contrast, the [Cu@Sn_9_]^3−^ anion shows an unusually sharp signal with a ^117^Sn–^119^Sn coupling constant of 85 Hz. No signals of protonated clusters [TM@Sn_9_H]^3−^ (TM = Ni and Pd), which are expected to be low field shifted by about 385 and 896 ppm in comparison to the filled cluster, were observed in our NMR experiments, despite the similar reaction conditions for their formation. Furthermore, these protonated [TM@Sn_9_H]^3−^ anions were proposed as intermediates towards larger oxidized clusters such as [Ni_2_@Sn_17_]^4−^, [Pd_2_@Sn_18_]^4−^ or to [Pt@Pb_12_]^4−^. In the present case the formation could not be observed by NMR or as crystallization products.^23,31,45,51^

For compound 7 one ^207^Pb signal at −3062 ppm is observed, showing a highfield shift of about 1000 ppm with respect to the peak of the unfilled [Pb_9_]^4−^ cluster. The peak shows satellites with a ^1^*J*(^195^Pt–^207^Pb) coupling constant of 4123 Hz. Generally, ^1^*J*(^195^Pt–^207^Pb) coupling constants are scarce in the literature, and the few reported ones for classical organometallics are in the range of 14.5 kHz and 18.5 kHz.^53^ However, in cluster anions the coupling constants are generally much shorter than in comparable organometallic molecules.^51^ Therefore, a much more precise comparison can be drawn between the coupling in the icosahedral anion [Pt@Pb_12_]^2−^ with a slightly lower ^1^*J*(^195^Pt–^207^Pb) of 3440 Hz.^51^ Analysis of the relative areas of the peak and satellite gives a ratio of 0.25 to 1.00, which is roughly the expected ratio based on the natural isotopic abundance of ^207^Pb to ^195^Pt.

In general, for *D*_3h_ symmetric clusters a splitting into two NMR signals with a ratio of 6 to 3 for prism atoms and capping atoms is expected, respectively. The absence of additional peaks in the −6000 to 6000 ppm window and the lack of Sn–Sn or Pb–Pb indicate that all atoms of the [TM@Sn_9_]^4−^ (TM = Ni or Pd) and [Pt@Pb_9_]^4−^ cluster are in fast exchange on the ^119^Sn/^207^Pb NMR time scale similar to for example [Cu@Sn_9_]^3−^. An exact mechanism of how the cluster surface atoms fluctuate around the central atom has not been proposed for filled nine-atom clusters before. We propose a similar mechanism to the diamond square rearrangement found in carboranes^54,55^ or [Tt_9_]^4−^.^56^ For unfilled clusters the energy barrier is extremely low (<4.7 kJ mol^−1^) between the cluster symmetry *D*_3h_ and *C*_4v_.^57,58^ Therefore, the atom movement *via* this mechanism would presumably be the easiest path way for endohedral clusters. This is further supported by the fact, that both “isomers” of [Ni@Sn_9_]^4−^ can be crystallized by varying the cations, indicating the presence of both symmetries at some point in the solution and a low energy difference between them.

### EPR spectroscopy

EPR spectroscopy was only feasible for compounds with unpaired electrons. Compounds 4–7 are diamagnetic and thus EPR silent, while compounds 1 and 2 decomposed rapidly preventing the acquisition of reliable EPR data.

For compound 3, based on the number of cations, a fourfold negative charge is assigned to the cluster anion [Co_0.827(5)_@Sn_9_]^4−^ (3a). The cluster [Co@Sn_9_]^4−^ has been previously been synthesized by the extraction of the ternary K–Co–Sn phase with en and was discussed as incorporating a cobalt atom.^17,30^ However, no clear proof for a closed or open shell d metal in the cluster anion [Co@Sn_9_]^4−^ has been given so far. To further investigate the spin population of compound 3, an EPR spectrum was measured. The EPR spectrum of 3 shows broad lines with a *g*_iso_ of 2.0581. The line broadening is a result of the hyperfine coupling (between Co and Sn) and makes the EPR lines appear isotropic. This observation indicates that the *g*_iso_ anisotropy of the system is low, suggesting that the spin density is located at the Co atom. An EPR spectrum of the related K[K(2,2,2crypt)]_3_[Co_0.870(4)_@Sn_9_] salt was reported by Sun *et al.*^17^ However, no g-value was provided, which makes a direct comparison with the EPR spectrum of compound 3 difficult.

**Fig. 5:**
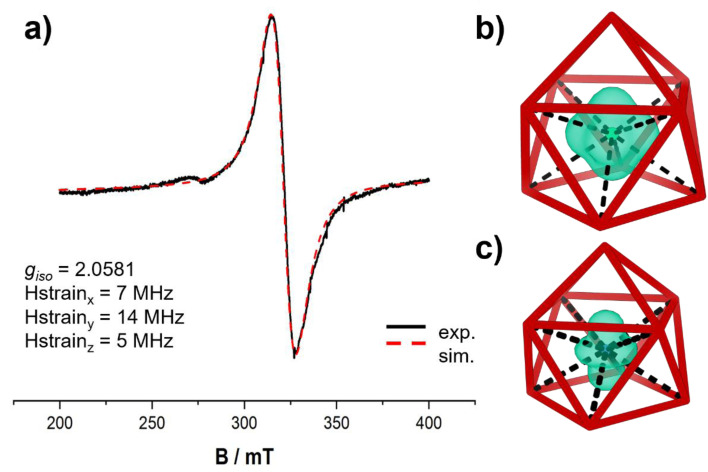
Simulated and experimental EPR spectra of anion 3a (a). Calculated spin density plots are given in (b) and (c). Around the central iron atom (b) and the central blue cobalt atom (c) the plotted spin density is shown in green. Isosurface level = 0.01. The cluster framework is simplified by red colored bonds.

The calculated Mulliken spin populations are 1.05 (3a) and 2.23 (1a), respectively. The reported spin populations were obtained from TPSSh single-point calculations on BPB86-optimized geometries, as hybrid functionals generally yield a more reliable description of spin localization. This is in line with the results of the EPR spectrum of compound 3. These findings indicate an almost exclusive localization of the unpaired electrons at the 3d metal centers as is also evident from inspection of the plotted spin densities (Fig. 5b and c).

Unlike earlier reports on the extraction of ternary K–Co–Sn phases,^14,15,17,30^ the present case does not proceed *via* such a pathway. Instead, two important aspects must be considered during the reaction. First, the ligands of the organometallic compounds have to be exchanged by the solvent or cluster to allow the cluster to incorporate the bare transition metal atom into its empty void. An intermediate for the successful attachment of the cluster anion to organometallic fragments like [(CO)_3_Cr(η^4^-Sn_9_)]^4−^ (ref. 43 and 59) has been previously reported. The formation of these species can be described by a ligand exchange reaction in the case of the replacement of neutral ligands (*e.g.* CO) at the transition metal atom through the *Zintl* cluster. Second, the oxidation state of the transition metal atom can change under the reductive conditions arising from the fourfold negatively charged nine-atomic clusters present in solution. For the group ten elements no change of the oxidation state is expected due to fully occupied d orbitals. The overall charge of the endohedral cluster is not changing during the reaction. However, for the earlier transition metals the electronic configuration is not always as clear. Therefore, the charge of the cluster can be either assigned as four times negative hosting a transition metal atom with partly filled d orbitals or a cluster with a reduced charge hosting an anionic transition metal atom with completely filled d orbitals. In the case of [Co(−i)@Sn_9_]^5−^ and [Ru(−ii)@Sn_9_]^6−^ the oxidation states have been assigned based on the charge of the cluster and quantum mechanical calculations. In the present case of compounds 1–3, the EPR spectrum of 3 together with the calculated Mulliken spin populations indicates the presence of zero-valent Co and Fe atoms with d^8^ and d^9^ electronic configurations inside the central cavity of the [Sn_9_]^4−^ cluster, with no change in the oxidation state during the reaction. Therefore, the reaction can be best described as a ligand exchange reaction. The organic ligands are substituted by the inorganic cluster, followed by opening of the cluster framework and migration of the transition metal atom from the cluster periphery towards the centre of the cluster unit. A similar reaction pathway has been proposed for the formation of [Cu@Sn_9_]^3−^, in which the intermediate [(iPr)Cu(η^4^-Sn_9_)]^3−^ has been isolated, and in which the oxidation state of the transition metal atom also remains unchanged.

## Conclusion

We successfully applied a facile synthesis for the formation of metalloid clusters. The clusters comprise tin and lead as surface atoms, and the elements range from Fe to Ni and from Ni to Pt as central atoms. The presence of the endohedral clusters in those compounds could be shown by single crystal structure determination, Raman and NMR spectroscopy. The NMR indicates a highly fluxional behaviour of the surface atoms around the central atom independent of the transition metal size. Further, the comparable structures show that the volume expansion of the clusters is highly related to the size of the central atom, but can also indicate different electronic configurations or bonding situations. EPR spectroscopy and DFT calculations support that the central Co atom is zero-valent with a d^9^ configuration.

The established synthetic approach provides a versatile entry point to a broader family of endohedral *Zintl* clusters, enabling systematic variation of the encapsulated atoms. We have extended the number of examples with Li counterions for which we have recently reported several salts with unfilled clusters. Here, we found that filled variants crystallize in many cases in the same isotypic structure. Thus, the degree of TM filling of the clusters might represent the ratio of filled and unfilled clusters in solution. In the crystals, filled and unfilled clusters of the same charge co-crystallize and reflect the statistical partial occupation of the clusters. The appearance of unfilled [Tt_9_]^4−^ and filled [TM@Tt_9_]^4−^ clusters is obvious for TM = Ni, Pd, and Pt^12,13,15^ with a d^10^ electron configuration, but requires changes in oxidation states for TM = Fe and Co leading to a deeper understanding of the variable amount of TM incorporation. This study opens pathways to explore how electronic structure and transition metal-tetrel interactions influence cluster reactivity. A deeper understanding of metalloid cluster chemistry will foster the targeted cluster expansion towards larger intermetalloid fused clusters and will contribute to the development of functional materials based on endohedrally filled clusters.

## Author contributions

C. E. F. and W. K. carried out the crystal structure determination by single crystal ray diffraction. C. E. F. wrote the manuscript draft. P.C. carried out the DFT computations and provided discussion. D. M. D. performed Raman experiments. T. F. F. contributed to project guidance, critical manuscript review, and funding acquisition.

## Conflicts of interest

There are no conflicts to declare.

## Supplementary Material

SC-OLF-D5SC08011H-s001

SC-OLF-D5SC08011H-s002

## Data Availability

Crystallographic data for compound 1–7 have been deposited at the Cambridge Crystallographic Data Centre (CCDC) under deposition numbers CCDC 2300165–2300171. CCDC 2300165–2300171 contain the supplementary crystallographic data for this paper.^60*a*–*g*^ The data supporting this article have been included as part of the supplementary information (SI). Supplementary information: NMR spectra, EDX analysis, complete range of measured Raman spectra, crystallographic details and selected bond lengths, and further experimental details. See DOI: https://doi.org/10.1039/d5sc08011h.
