# Supplementary material for: Endohedral anionic nine-atom Zintl clusters of the elements tin and lead with lithium counterions
Source: Chem Sci. 2026 Jan 9;17(11):5498–509. doi: 10.1039/d5sc08011h (PMC12826108; doi:10.1039/d5sc08011h)
Supplement: SC-017-D5SC08011H-s001 [file SC-017-D5SC08011H-s001.pdf]

Supplementary Information (SI) for Chemical Science.
This journal is © The Royal Society of Chemistry 2026

Supporting Information

Endohedral Anionic Nine-Atom Zintl Clusters of the Elements Tin and Lead with Lithium Counterions

C.E. Fajman,[a] D. M. Dankert,[a] P. Coburger,[a] W. Klein,[b] and T. F. Fässler[a]*

[a] Technical University of Munich, TUM School of Natural Sciences, Chemistry Department, Chair of Inorganic Chemistry with Focus on New Materials, Lichtenbergstraße 4, 85748 Garching, Germany

[b] Technical University of Munich, Catalysis Research Centre, Ernst-Otto-Fischer-Straße 1, 85748 Garching, Germany

Content

1. Experimental details 2
2. NMR 7
3. EDX 10
4. Raman 15
5. PXRD 16
5. Volume change 17
6. Crystallographic details 19
7. Cartesian coordinates of optimized structures 31
7. References 32

1. Experimental details

General

All manipulations were performed under the exclusion of moisture and oxygen by utilizing standard Schlenk and glove box techniques under Argon atmosphere. Ethylenediamine was distilled over CaH_2, all other solvents were dried by a MBraun Grubbs solvent purification system and subsequently degassed by freeze-pump-thaw. Glassware was dried at 650 °C under dynamic vacuum prior to usage. Anhydrous LiCl was dried at 500°C by applying dynamic vacuum for 8 h. The solid-state precursors K_4Tt_9 (Tt = Sn, Pb) were prepared by a slightly modified literature procedure.[1, 2] The elements were heated with a rate of 2 °C/h in stainless-steel autoclaves and fused at 550 °C and 650 °C for 48 h, respectively. Followed by a slow cooling at a rate of - 1°C/h. All other chemicals were used without further purification. $Ni(COD)_2$, $TM(PPh_3)_4$ were obtained by Merck.

Single crystal structure determination

The moisture- and air-sensitive crystals were transferred into perfluoroalkyl ether oil in a glove box. Suitable single crystals were isolated under a microscope and fixed on glass capillaries. The data collections were performed using a STOE StadiVari diffractometer (Mo K_α radiation) equipped with a DECTRIS PILATUS 300K detector, with the crystal positioned in a 150 K cold N_2 gas stream. Direct methods (SHELXS-97)[3] were used to solve the structures, and the structure refinements were performed by full-matrix least-squares calculations against F^2 (SHELXL-2018).[4] Anisotropic displacement parameters were set for all non-hydrogen atoms except for Fe in compound **1**, because during the anisotropic refinement of this Fe position the displacement parameters become negative. The positions of hydrogen atoms were modelled using a riding model.

NMR spectroscopy

Compounds **4**, **5** and 7 were dissolved in *en* and the NMR tube equipped with a lock capillary filled with C_6D_6. ^{7}Li NMR, ^{119}Sn NMR and ^{207}Pb NMR spectra were acquired on a Bruker Avance Ultrashield 400 MHz or a Bruker Avance 300 spectrometer. ^{7}Li NMR, ^{119}Sn NMR and ^{207}Pb spectra were calibrated by an external standard (9.7 M LiCl in D_2O, Me_4Sn 90% in C_6D_6, and $Pb(NO_3)_2$ in D_2O).

Energy Dispersive X-Ray (EDX) Analysis

For the data collection a Hitachi TM-1000 tabletop scanning electron microscope equipped with an energy dispersive X-ray analyzer was used. The respective samples were stuck on a carbon sticky tape under Ar atmosphere, and the samples were transferred to the EDX device in a closed glass. Immediately before insertion, the

container was opened and the probe was transferred as quickly as possible to minimize air exposure. Prior to the measurement he EDX Detector was calibrated with a Cu-Standard (piece of copper tape).”

EPR spectroscopy

EPR measurements were carried out using a JEOL JES-FA 200 spectrometer at X-band frequency (approximately 9.05 GHz, sweep width 50 mT, modulation frequency 100 kHz, modulation amplitude 0.4 mT, microwave power 5.0 mW, measurement temperature 120 K). The *g* values were determined using Mn^{2+} (nuclear spin I = 5/2) embedded in MgO as a standard (fourth line g = 1.981). The temperature was monitored with a JEOL DVT4 temperature controller. The measurement was performed on solid crystalline sample. The samples were cooled to N_2(l) temperature prior to the measurements. Simulation of EPR spectra occurred using the simulation program Easyspin.[5]

Raman

Raman spectroscopy was performed using a Renishaw inVia Raman microscope equipped with a CCD detector and 520 nm lasers with a maximum power of 0.01 mW. The software WiRe 4.2 (build 5037, Renishaw 2002) was used for operating the device. All samples were measured on single crystals in glass capillaries (outer diameter 0.3 mm, wall thickness 0.01 mm, MARK capillaries, Müller & Müller OHG), which were sealed using capillary wax (Hampton Research).

Volume calculations

The volume of the nine-atomic clusters were calculated using the program polynator.[6] The central *TM* atom has been set as the central atom with a radius of 3 Å, while the framework atoms of the cluster have been assigned as the ligand atoms (Sn or Pb). The additional data which are not reported in the present work have been obtained from CCDC. References see Table S9.

Computational Details

All calculations were carried out with the ORCA program package.[7, 8] To balance the high negative charge of the investigated anions, the CPCM model was used for all calculations setting the dielectric constant of the embedding medium to infinity. Density fitting techniques, also called resolution-of-identity approximation (RI)[9], were used for geometry optimisations at the BP86-D3BJ/def2-SVP level and the RIJCOSX[10] approximation was used for calculating the spin density at the TPSS0/cc-pVTZ-DK[11-13] level.

Powder X-ray Diffraction

For powder X-ray diffraction (PXRD) analysis the samples were finely ground in an agate mortar, diluted with diamond powder and sealed in glass capillaries (outer diameter 0.3 mm, wall thickness 0.01 mm, MARK capillaries, Müller & Müller OHG) with capillary wax (Hampton Research) in an argon-filled glove box. PXRD were performed at room temperature by using a Stoe STADI P powder diffractometer equipped with a linear position-sensitive detector (Mythen 1K) using Cu $K_{\alpha 1}$ (λ = 1.54060 Å) and curved Ge (111) monochromators. The samples were measured in Debye-Scherrer geometry ($2\theta_{max}$ = 22.5°). Data analysis was carried out by using Stoe WinXPOW software package.[14]

Syntheses

$[Li(en)_2]_4[Fe_{0.059(3)}@Sn_9]$ (1)

In a first step the *Zintl* phase K_4Sn_9 (91.9 mg, 75 µmol, 1 equiv.) and IPrFeDVTMS (47.3 mg, 75 µmol, 1 equiv.) are added to a Schlenk tube and 2 mL en are added. The mixture is left to stir for 1 h. Subsequently, the solution is added to a flask loaded with LiCl (12.7 mg, 300 µmol, 4 equiv.) and the reaction mixture stirred for 3 h at r.t. Afterwards the solution is filtered and the deeply red colored filtrate is layered with 2 ml of toluene. After 3 days red plates have grown on the wall of the Schlenk tube.

EPR silent. **Raman** 156 cm^{-1}, 170 cm^{-1}. **EDX**: Fe 2.2% (calc. 0.3%), Sn 97.8% (calc. 99.7%).

$[K[18]crown\text{-}6]_2K_2[Fe_{0.055(4)}@Sn_9] \cdot 1.5$ en (2)

The *Zintl* phase K_4Sn_9 (91.9 mg, 75 µmol, 1 equiv.), IPrFeDVTMS (47.3 mg, 75 µmol, 1 equiv.) and 18-crown-6 (39.6 mg, 150 µmol, 1 equiv.) are weighted into a Schlenk tube and 2 mL en are added. The mixture is left to stir for 3 h. Subsequently, the solution is filtered and the deeply red colored filtrate is layered with 2 ml of toluene. After 3 days red needles have grown on the wall of the Schlenk tube.

EPR silent. **Raman** 154 cm^{-1}, 168 cm^{-1}. **EDX**: K 12.7% (calc. 12.7%), Fe 2.2% (calc. 0.3%), Sn 85.0% (calc. 87.0%).

$[Li(en)_2]_4[Co_{0.827(5)}@Sn_9]$ (3)

In a first step the *Zintl* phase K_4Sn_9 (91.9 mg, 75 µmol, 1 equiv.) and IPrCoDVTMS (47.5 mg, 75 µmol, 1 equiv.) are added to a Schlenk tube and 2 mL en are added. The mixture is left to stir for 1 h. Subsequently, the solution is added to a flask loaded with LiCl (12.7 mg, 300 µmol, 4 equiv.) and the reaction mixture stirred for 3 h at r.t. Afterwards the solution is filtered and the deeply red colored filtrate is layered with 2

ml of toluene. After 7 days thin and red plates have grown on the wall of the Schlenk tube.

EPR g_{iso} = 2.0581**.** **Raman** 156 cm^{-1}, 168 cm^{-1}. **EDX**: Co 4.1% (calc. 4.4%), Sn 95.9% (calc. 95.6%).

$[Li(en)_2]_4[Ni@Sn_9]$ (4)

In a first step the *Zintl* phase K_4Sn_9 (91.9 mg, 75 µmol, 1 equiv.) and $Ni(COD)_2$ (20.6 mg, 75 µmol, 1 equiv.) are added to a Schlenk tube and 2 mL en are added. The mixture is left to stir for 1 h. Subsequently, the solution is added to a flask loaded with LiCl (12.7 mg, 300 µmol, 4 equiv.) and the reaction mixture stirred for 3 h at r.t. Afterwards the solution is filtered and the deeply red colored filtrate is layered with 2 ml of toluene. After 7 days red plates have grown on the wall of the Schlenk tube.

^{7}Li NMR (300 MHz, 298 K, en/C_6D_6): δ [ppm] = 1.54 ppm. **^{119}Sn NMR** (300 MHz, 298 K, en/C_6D_6): δ [ppm] = 1232 ppm (< 158 Hz). **Raman** 149 cm^{-1}, 168 cm^{-1}. **EDX**: Ni 5.1% (calc. 5.2%), Sn 94.9% (calc. 94.8%).

$[Li(en)_2]_4[Pd_{0.824(4)}@Sn_9]$ (5)

In a first step the *Zintl* phase K_4Sn_9 (91.9 mg, 75 µmol, 1 equiv.) and $Pd(PPh_3)_4$ (86.7 mg, 75 µmol, 1 equiv.) are added to a Schlenk tube and 2 mL en are added. The mixture is left to stir for 1 h. Subsequently, the solution is added to a flask loaded with LiCl (12.7 mg, 300 µmol, 4 equiv.) and the reaction mixture stirred for 3 h at r.t. Afterwards the solution is filtered and the deeply red colored filtrate is layered with 2 ml of toluene. After 3 days red plates have grown on the wall of the Schlenk tube.

^{7}Li NMR (300 MHz, 298 K, en/C_6D_6): δ [ppm] = 1.66 ppm. **^{119}Sn NMR** (300 MHz, 298 K, en/C_6D_6): δ [ppm] = 763 ppm (74 < Hz). **Raman** 154 cm^{-1}, 170 cm^{-1}. **EDX**: Pd 5.9% (calc. 7.6%), Sn 94.1% (calc. 92.4%).

$[Li(en)_2]_4[Pd_{0.460(5)}@Pb_9]$ (6)

In a first step the *Zintl* phase K_4Pb_9 (91.9 mg, 75 µmol, 1 equiv.) and $Pd(PPh_3)_4$ (86.7 mg, 75 µmol, 1 equiv.) are added to a Schlenk tube and 2 mL en are added. The mixture is left to stir for 1 h. Subsequently, the solution is added to a flask loaded with LiCl (12.7 mg, 300 µmol, 4 equiv.) and the reaction mixture stirred for 3 h at r.t. Afterwards the solution is filtered and the deeply red colored filtrate is layered with 2 ml of toluene. After 3 days red plates have grown on the wall of the Schlenk tube.

Raman: no bands were observed. **EDX**: Pd 4.3% (calc. 2.6%), Sn 95.7% (calc. 97.4%).

$[Li(en)_2]_4[Pt_{0.426(4)}@Pb_9]$ (7)

In a first step the *Zintl* phase K_4Pb_9 (91.9 mg, 75 µmol, 1 equiv.) and $Pt(PPh_3)_4$ (93.3 mg, 75 µmol, 1 equiv.) are added to a Schlenk tube and 2 mL en are added. The mixture is left to stir for 1 h. Subsequently, the solution is added to a flask loaded with LiCl (12.7 mg, 300 µmol, 4 equiv.) and the reaction mixture stirred for 3 h at r.t. Afterwards the solution is filtered and the deeply red colored filtrate is layered with 2 ml of toluene. After 14 days tiny red plates besides a metallic dust have grown on the wall of the Schlenk tube. Crystallization and reaction experiments have been done under the exclusion of light and at lower temperatures (6°C). However, only under the complete exclusion of light was it possible to obtain single crystals, therefore we conclude that the compound is light sensitive.

^{7}Li NMR (300 MHz, 298 K, en/C_6D_6): δ [ppm] = 1.7. **^{209}Pb NMR** (300 MHz, 298 K, en/C_6D_6): δ [ppm] = -3062 ppm ($^{1}J(^{195}Pt-^{207}Pb)$ = 3440 Hz). **Raman:** no bands were observed. **EDX**: Pt 7.6% (calc. 4.6%), Pb 92.4% (calc. 95.8%).

2. NMR

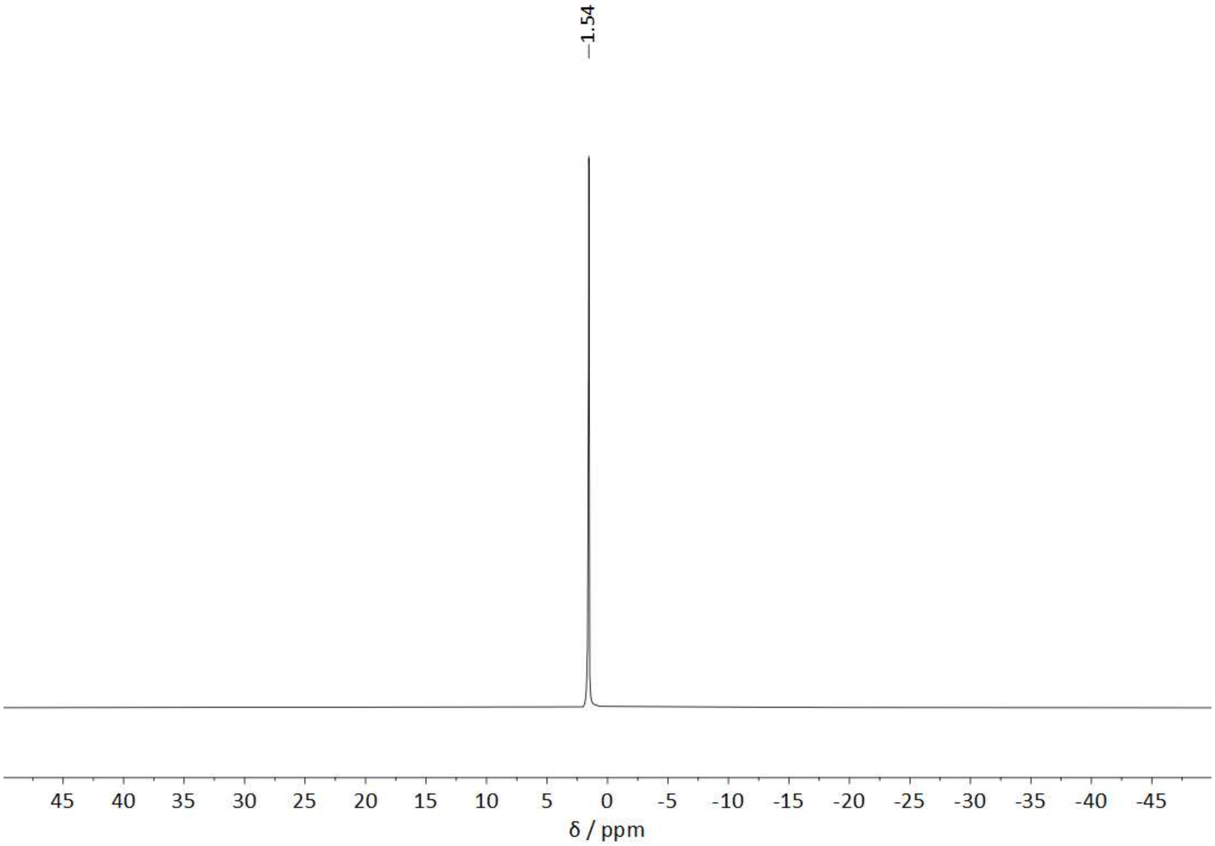

Figure S1: ^{7}Li NMR spectrum of compound **4** recorded from *en* with a C_6D_6 lock capillary.

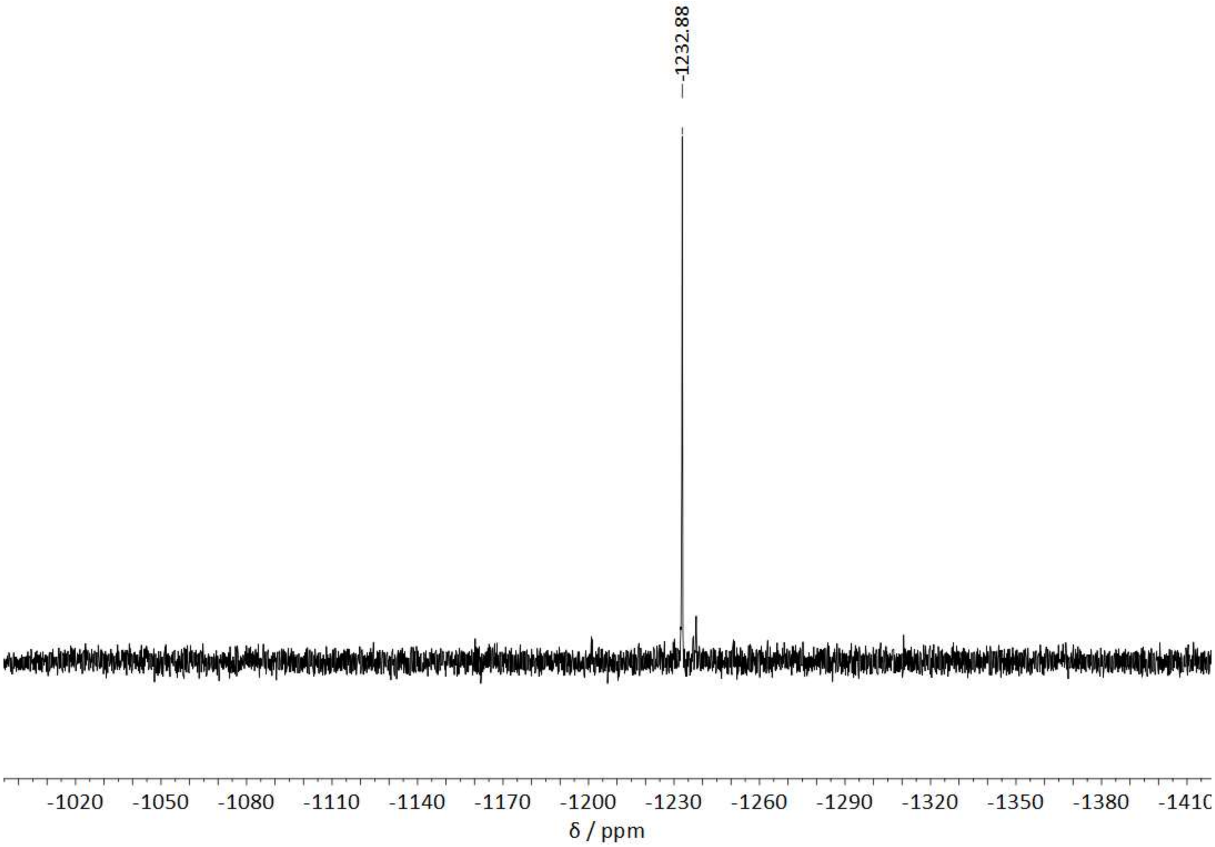

Figure S2: ^{119}Sn NMR spectrum of compound **4** recorded from *en* with a C_6D_6 lock capillary.

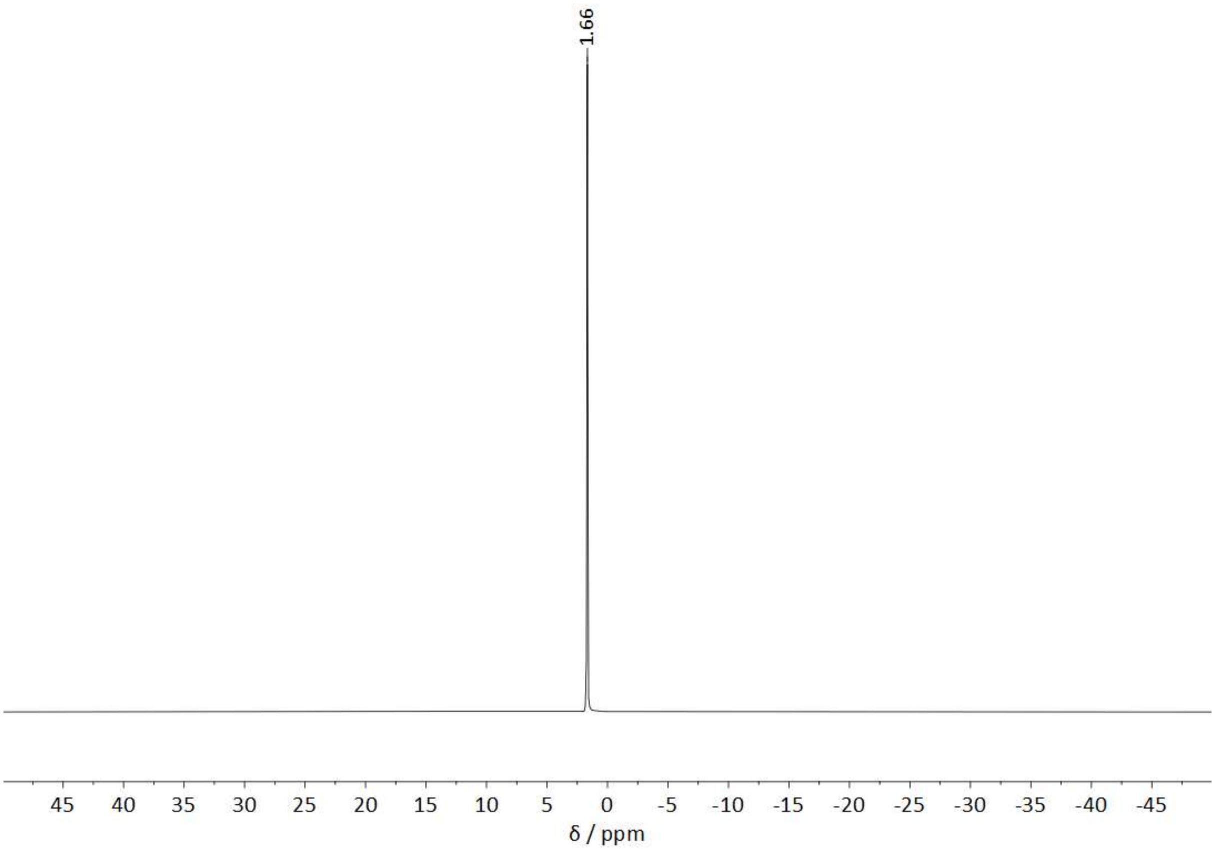

Figure S3: ^{7}Li NMR spectrum of compound **5** recorded from *en* with a C_6D_6 lock capillary.

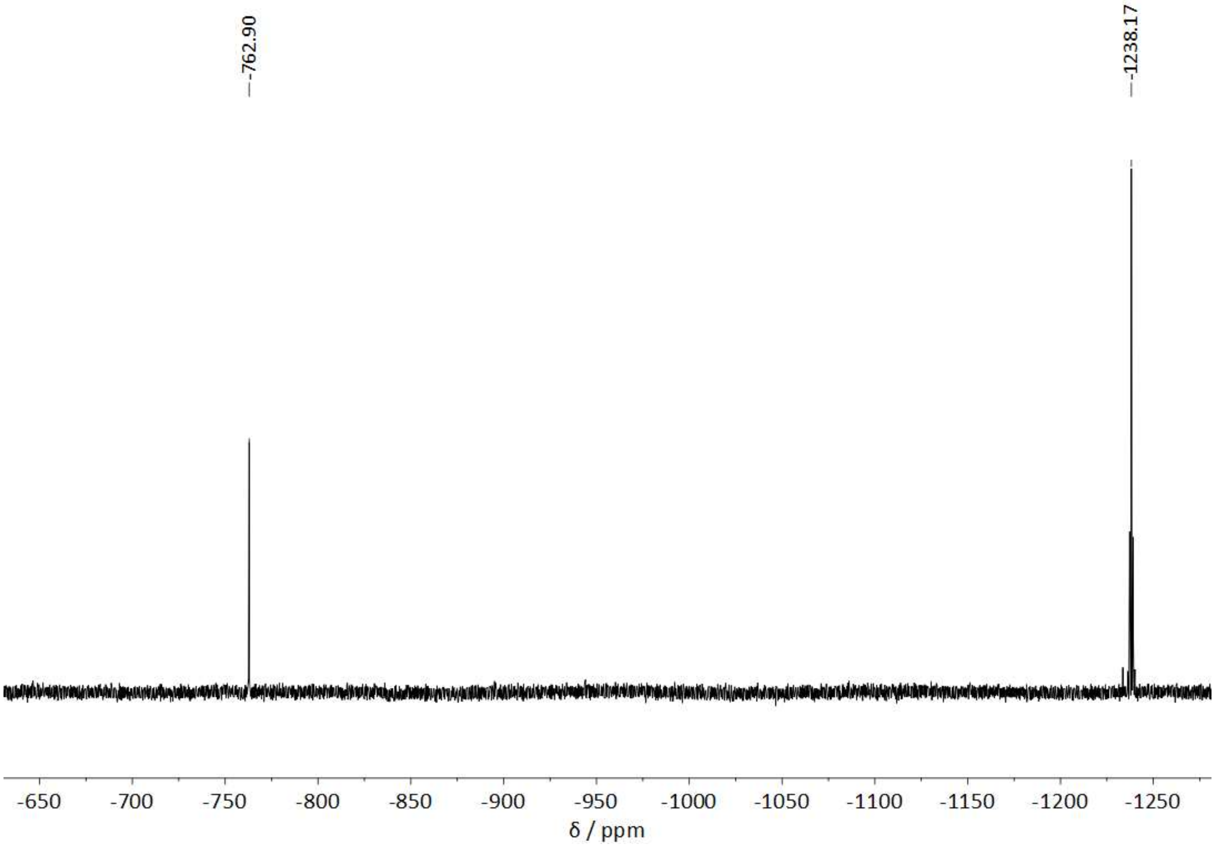

Figure S4: ^{119}Sn NMR spectrum of compound **5** recorded from *en* with a C_6D_6 lock capillary.

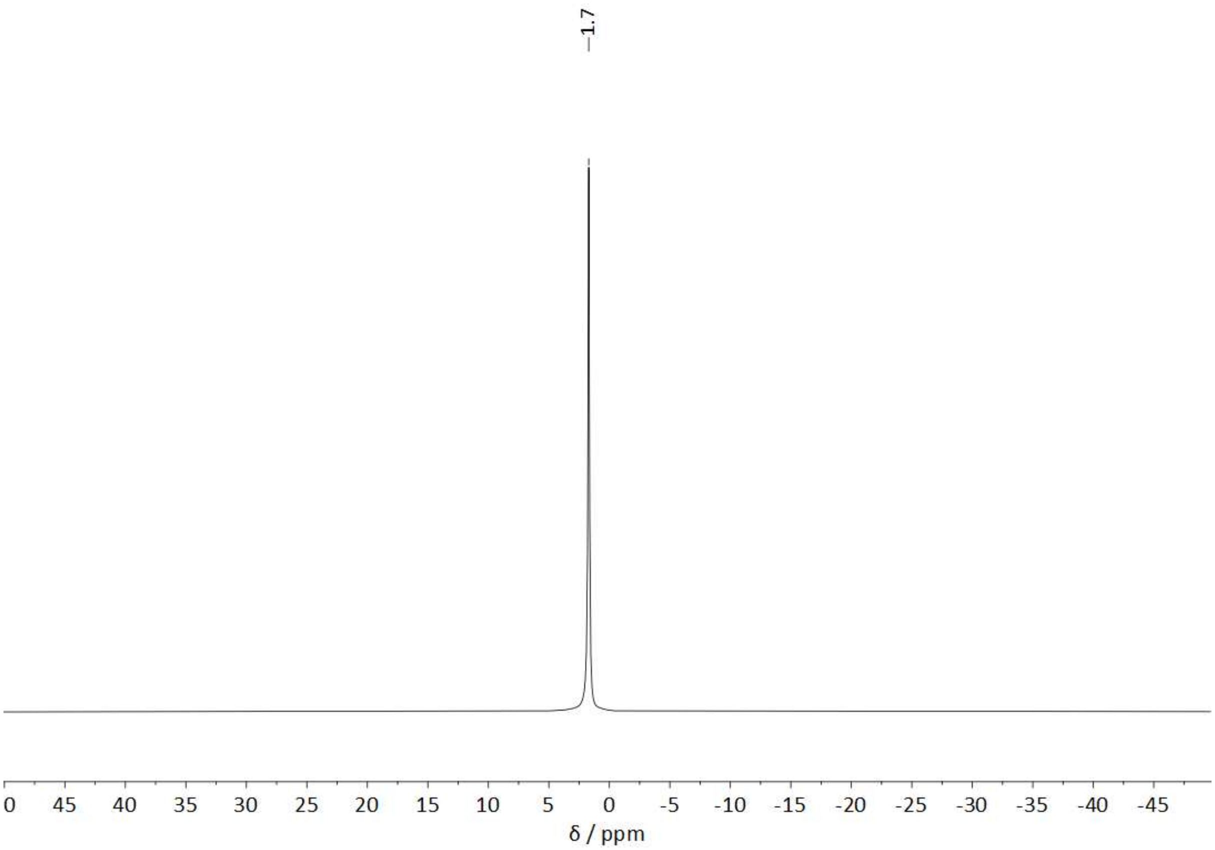

Figure S5: ^{7}Li NMR spectrum of compound **7** recorded from *en* with a C_6D_6 lock capillary.

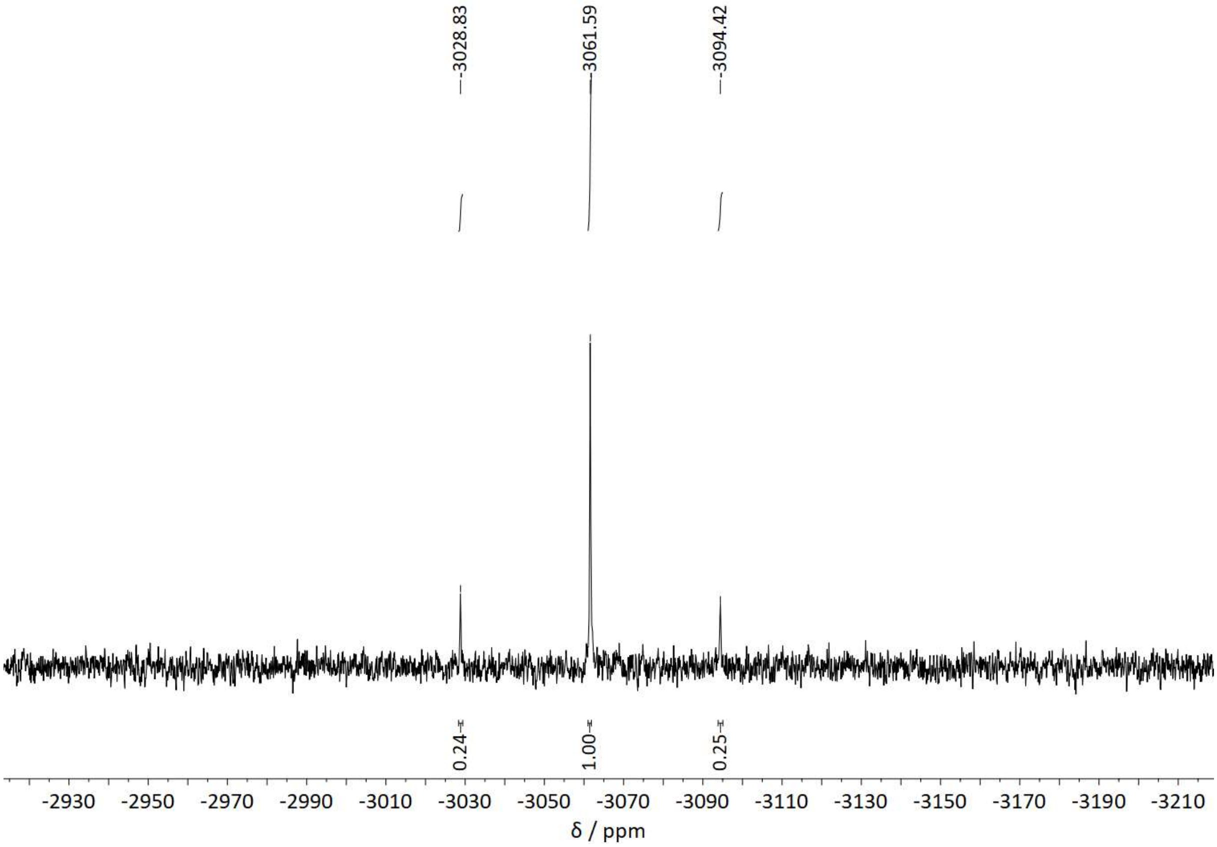

Figure S6: ^{207}Pb NMR spectrum of compound **7** recorded from *en* with a C_6D_6 lock capillary.

3. EDX

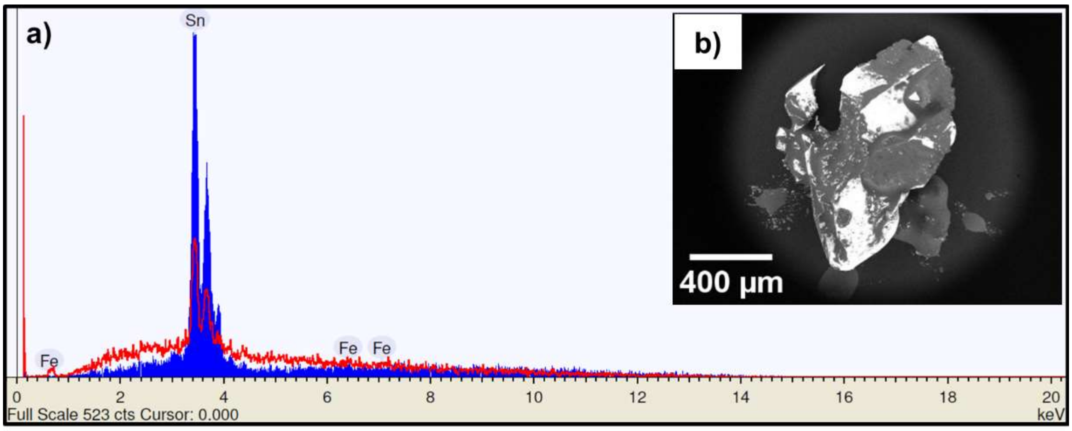

Figure S7: EDX analysis of **1** (a) on a broken single crystal, which is shown as a SEM microimage (b). The single crystal broke during the transfer to the sample holder.

Table S1: EDX analysis of compound **1**.

Elements	mass percent (exp.) / wt%	mass percent (calc.) / wt%	atom percent (exp.) / wt%	atom percent (calc.) / wt%
Fe	2.2%	0.3%	4.6%	0.7%
Sn	97.8%	99.7%	95.4%	99.3%

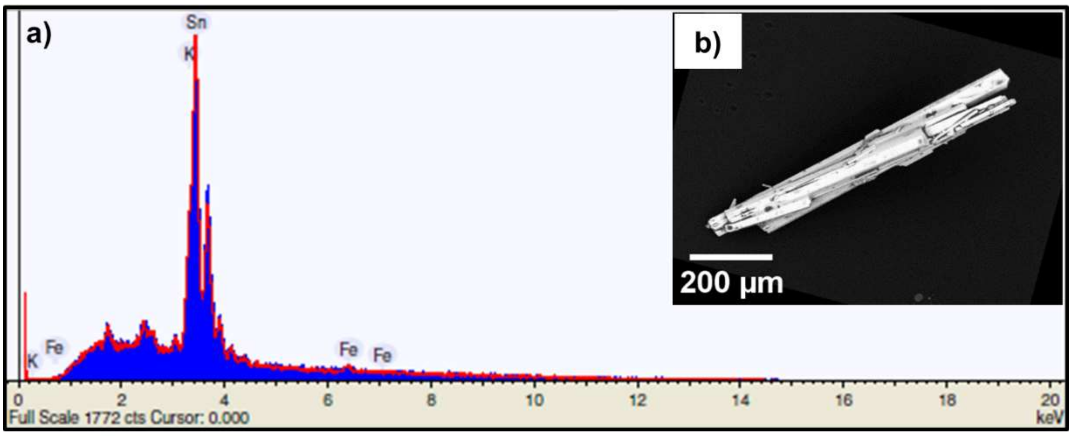

Figure S8: EDX analysis of **2** (a) on single crystals, which are shown as a SEM microimage (b).

Compound **2** crystallized as needles, which easily break therefore a needle pile was transferred to the sample holder.

Table S2: EDX analysis of compound **2**.

Elements	mass percent (exp.) / wt%	mass percent (calc.) / wt%
**K**	12.7%	12.7%
**Fe**	2.2%	0.3%
**Sn**	85.0%	87.0%

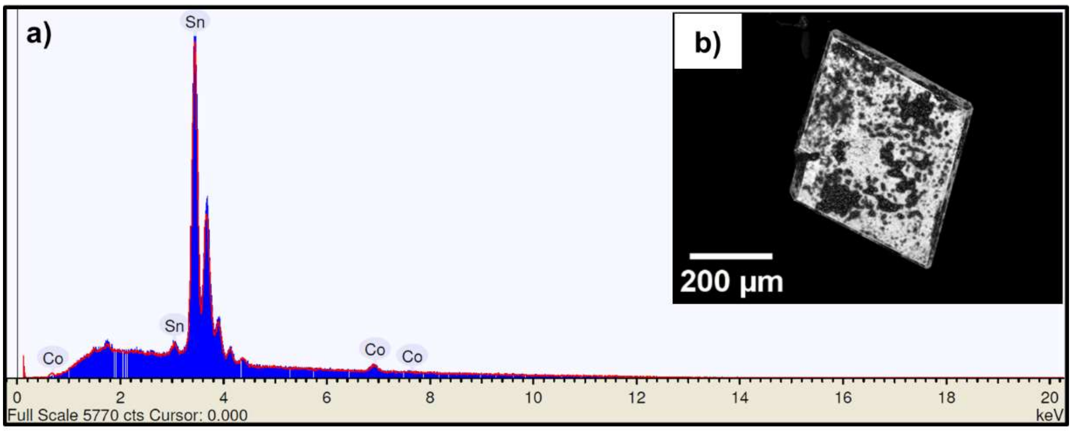

Figure S9: EDX analysis of **3** (a) on a single crystal, which is shown as a SEM microimage (b).

Table S3: EDX analysis of compound **3**.

Elements	mass percent (exp.) / wt%	mass percent (calc.) / wt%	atom percent (exp.) / wt%	atom percent (calc.) / wt%
**Co**	4.1%	4.4%	7.9%	8.4%
**Sn**	96.0%	95.6%	92.1%	91.6%

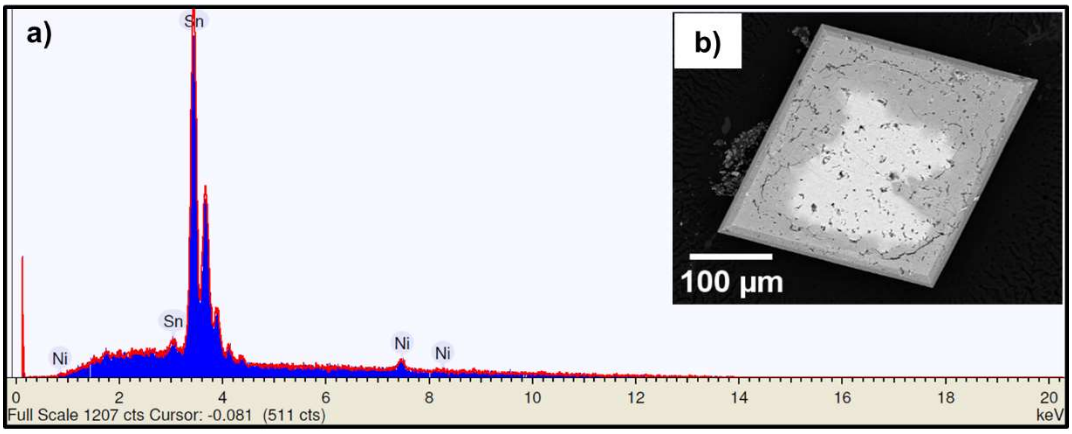

Figure S10: EDX analysis of **4** (a) on a single crystal, which is shown as a SEM microimage (b).

Table S4: EDX analysis of compound **4**.

Elements	mass percent (exp.) / wt%	mass percent (calc.) / wt%	atom percent (exp.) / wt%	atom percent (calc.) / wt%
**Ni**	5.1%	5.2%	9.8%	10.0%
**Sn**	94.9%	94.8%	90.2%	90.0%

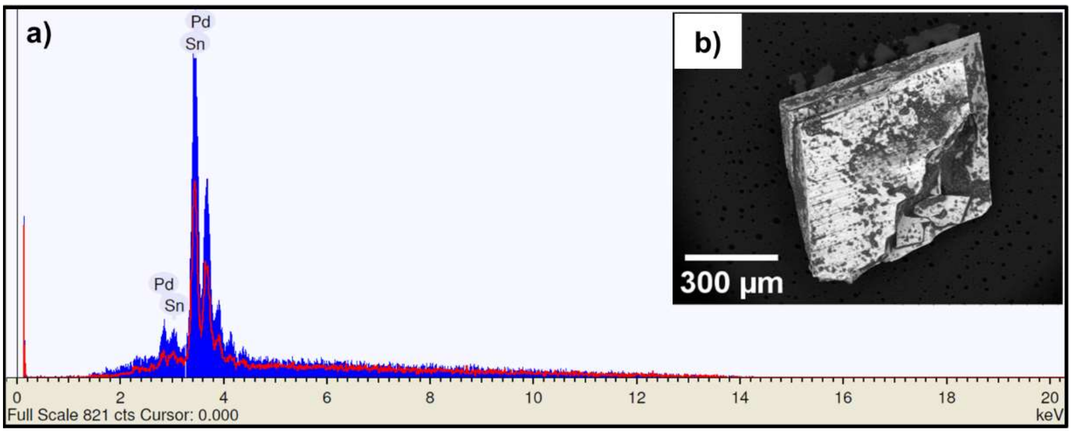

Figure S11: EDX analysis of **5** (a) on a broken single crystal, which is shown as a SEM microimage (b).

Table S5: EDX analysis of compound **5**.

Elements	mass percent (exp.) / wt%	mass percent (calc.) / wt%	atom percent (exp.) / wt%	atom percent (calc.) / wt%
**Pd**	5.9%	7.6%	6.5%	8.4%
**Sn**	94.1%	92.4%	93.5%	91.6%

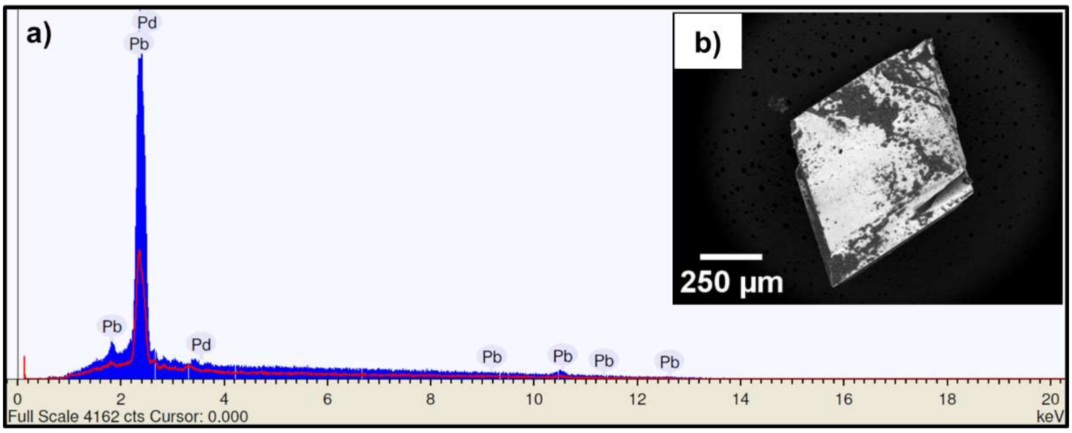

Figure S12: EDX analysis of **6** (a) on a single crystal, which is shown as a SEM microimage (b).

Table S6: EDX analysis of compound **6**.

Elements	mass percent (exp.) / wt%	mass percent (calc.) / wt%	atom percent (exp.) / wt%	atom percent (calc.) / wt%
**Pd**	4.3%	2.6%	8.0%	4.9%
**Pb**	95.7%	97.4%	92.0%	95.1%

According to ^{207}Pb NMR analysis of a reaction solution of compound **7** no peak besides the endohedral cluster was found. Therefore, also in single crystals of compound **7** no empty cluster should be present. However, the single crystal refinements result in an occupation of the Pt atom position of only 42.6 %, which indicates a decomposition during the crystallization. Compound **7** only crystalized in small quantities as tiny dark red single crystals besides large quantities of metallic powder. In the SEM microimage the metallic powder can be identified as small needles, which are more platinum rich than the single crystals. Therefore, we would assign the metallic needles as a decomposition product of the cluster anion **7a**.

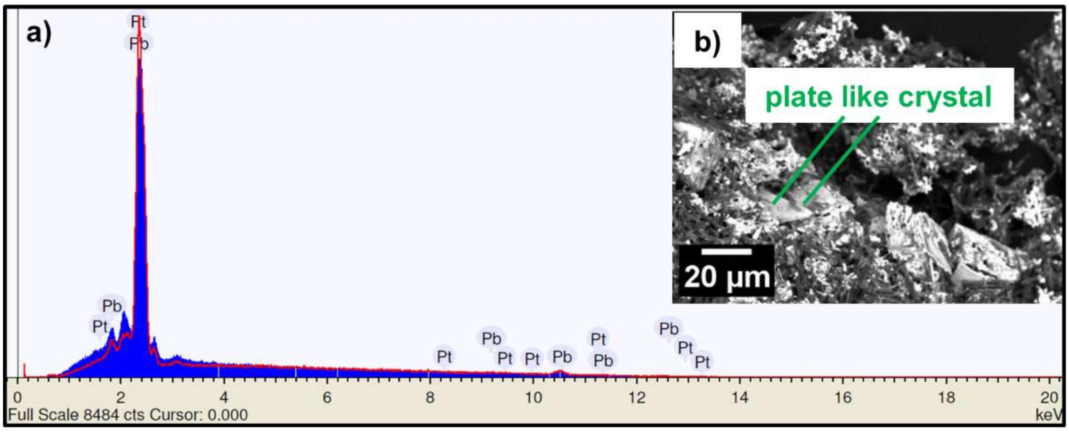

Figure S13: EDX analysis of **7** (a) on a single crystal in a pile of fine metallic needles, which is shown as a SEM microimage (b).

Table S7: EDX analysis of compound **7**.

Elements	mass percent (exp.) / wt%	mass percent (calc.) / wt%	atom percent (exp.) / wt%	atom percent (calc.) / wt%
**Pt**	7.6%	4.2%	8.0%	4.5%
**Pb**	92.4%	95.8%	92.0%	95.5%

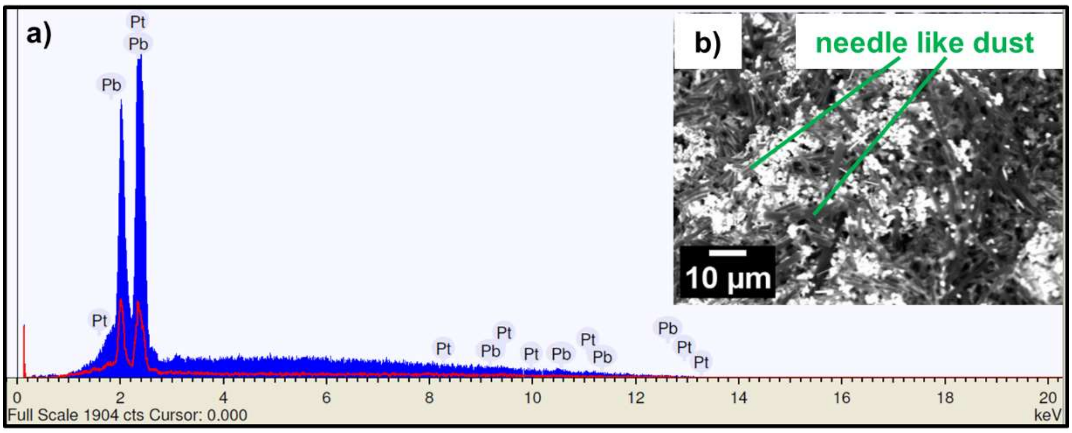

Figure S14: EDX analysis of the metallic powder, which precipitated as fine needles during the crystallization of compound **7** (a). The needles are shown as a SEM microimage (b).

Table S8: EDX analysis of the metallic needle like precipitate besides the single crystals of compound **7**.

Elements	mass percent (exp.) / wt%	atom percent (exp.) / wt%
**Pt**	23.0%	24.1%
**Pb**	77.00	75.9%

4. Raman

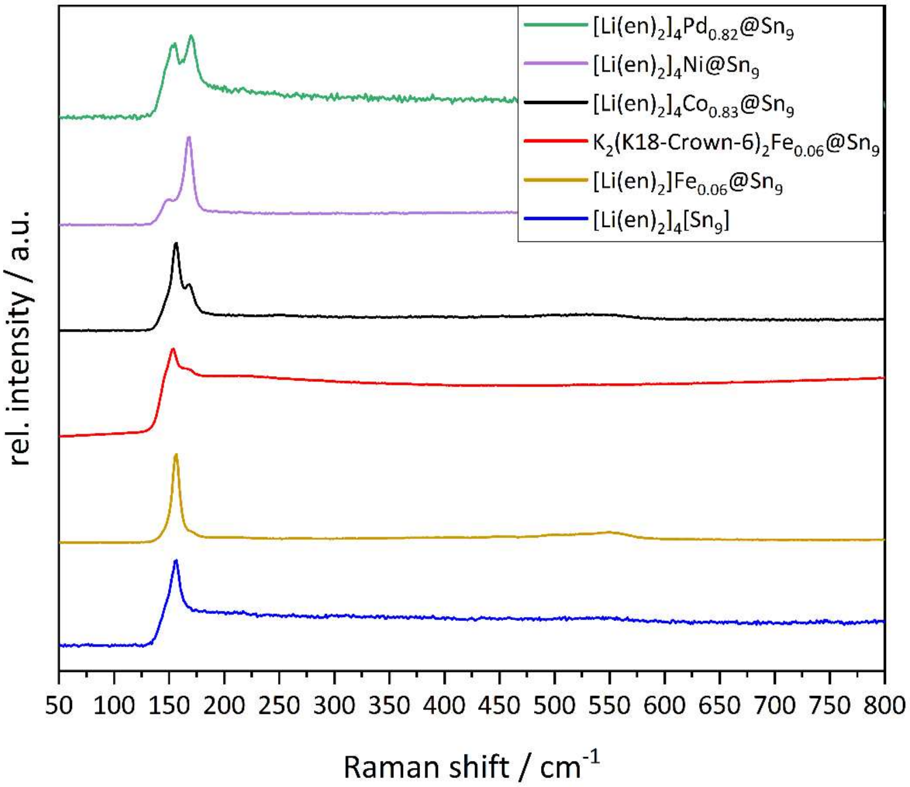

Figure S15: Raman spectra measured on single crystals of compound **1** to **5** and of the unfilled cluster as a reference.

5. PXRD

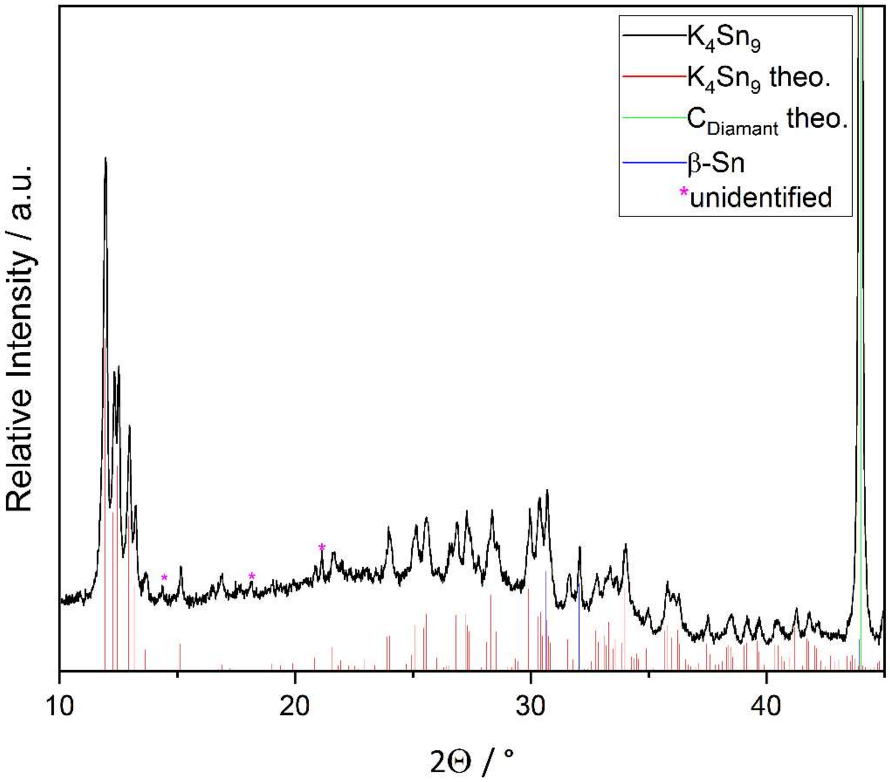

Figure S16: Powder diffractogram of synthesized K_4Sn_9. Diamond powder was used to dilute K_4Sn_9 prior to the measurement. The intensive reflection of diamond was truncated in the figure to improve the visibility of relevant reflections.

5. Volume change

Table S9: Calculated cluster volumes, difference of volume to the empty cluster, volume increase and normalized volume increase are shown with their respective occupancy**.** Crystal structure data for the calculation of the cluster volume were taken from Ref.[15-21]

**compound**	**cluster**	**cluster volume (V_c) [Å^3]**	**ΔV_c [Å^3]**	**volume increase [%]**	**normalized volume increase [%]**	**occupancy [%]**
$[Li_4(en)_8][Fe_{0.059(3)}@Sn_9]$	$[Fe@Sn_9]^{4-}$ (**1a**)	33.2	0.2	0.5	8.1	5.9
$[K[18]crown\text{-}6]_2K_2$ $[Fe_{0.055(4)}@Sn_9]$ · 1.5 en	$[Fe@Sn_9]^{4-}$ (**2a**)	33.2	0.2	0.6	11.2	5.5
$[Li_4(en)_8][Co_{0.827(5)}@Sn_9]$	$[Co@Sn_9]^{4-}$ (**3a**)	36.0	3.0	9.1	11.0	83.0
$K[K([2.2.2]crypt)]_3[Co_{0.68}@Sn_9]$	$[Co@Sn_9]^{4-}$	35.4	2.4	7.3	10.8	67.6
$K[K([2.2.2]crypt)]_3[Co_{0.87}@Sn_9]$	$[Co@Sn_9]^{4-}$	36.1	3.1	9.4	10.8	87.0
$[K([2.2.2]crypt)]_3$ $[Co@Sn_9Ni(CO)]$ · DMF	$[Co@Sn_9Ni(CO)]^{3-}$	36.6	3.6	10.8	10.8	100.0
$[K([2.2.2]crypt)]_3$ $[Co@Sn_9Ni(C_2H_4)]$ · en	$[Co@Sn_9Ni(C_2H_4)]^{3-}$	36.3	3.3	10.1	10.1	100.0
$[K([2.2.2]crypt)]_6$ $[Co@Sn_9Pt(PPh_3)]_2$	$[Co@Sn_9Pt(PPh_3)]^{4-}$	37.3	4.3	12.9	12.9	100.0
$[K([2.2.2]crypt)]_3$ [Co@Sn9AuPh]	$[Co@Sn_9AuPh]^{4-}$	37.2	4.2	12.7	12.7	100.0
$K_5Co_{1-x}Sn_9$	$[Co(-I)@Sn_9]^{5-}$	-	-	12.1	14.8	82.0
$K_{13}Co_{1-x}Sn_{17}$	$[Co(-I)@Sn_9]^{5-}$	-	-	14.6	14.6	100.0
$[Li_4(en)_8][Ni@Sn_9]$	$[Ni@Sn_9]^{4-}$ (**4a**)	36.6	3.6	10.9	10.9	100.0
$Na_{12}Ni_{1-x}Sn_{17}$	$[Ni@Sn_9]^{4-}$-I	36.2	3.2	9.6	10.1	95.0
	$[Ni@Sn_9]^{4-}$-II	36.2	3.2	9.7	10.5	92.6
$[K([18]crown\text{-}6)]_3[KSn_9]$ · 3 C_6H_6	$[Ni@Sn_9]^{4-}$	34.8	1.8	5.4	9.0	60.2
$[K([2.2.2]crypt)]_3[Ni@Sn_9]$	$[Ni@Sn_9]^{4-}$	36.1	3.1	9.5	9.5	100.0
$[K([2.2.2]crypt)]_6[(Ni@Sn_9)_2Cd]$ · en	$[(Ni@Sn_9)_2Cd]^{6-}$	36.1	3.1	9.4	9.4	100.0
$[K([2.2.2]crypt)]_5[(Ni@Sn_9)_2In]$ · DMF	$[(Ni@Sn_9)_2In]^{5-}$	35.6	2.6	7.7	7.7	100.0
$[K([2.2.2]crypt)]_3[Cu@Sn_9]$ · 2 DMF	$[Cu(+I)@Sn_9]^{3-}$	37.3	4.3	12.9	12.9	100.0
$[K_7(OH)][Ru@Sn_9]$ · 10 NH_3	$[Ru(-II)@Sn_9]^{6-}$	-	-	17.9	17.9	100.0
$[Li_4(en)_8][Pd_{0.824(4)}@Sn_9]$	$[Pd@Sn_9]^{4-}$ (**5a**)	38.7	5.7	17.3	21.1	82.0
$K_{12}Pd_{1-x}Sn_{17}$	$[Pd@Sn_9]^{4-}$ -I	36.0	3.0	9.1	16.5	55.5
	$[Pd@Sn_9]^{4-}$ -II	36.2	3.2	9.7	18.0	54.2
	$[Pd@Sn_9]^{4-}$ -III	34.0	1.0	3.1	11.9	26.1
$[K([2.2.2]crypt)]_3[Cu@Pb_9]$ · 2 DMF	$[Cu(+I)@Pb_9]^{3-}$	41.7	3.0	9.0	9.0	100.0
K_4RhPb_9	$[Rh@Pb_9]^{4-}$ -I	43.3	4.6	12.0	12.0	100.0
	$[Rh@Pb_9]^{4-}$ -II	44.0	5.3	13.8	13.8	100.0
$[Li_4(en)_8][Pd_{0.460(5)}@Pb_9]$	$[Pd@Sn_9]^{4-}$ (**6a**)	40.6	1.9	5.7	12.4	46.0
$[Li_4(en)_8][Pt_{0.426(4)}@Pb_9]$	$[Pt@Sn_9]^{4-}$ (**7a**)	40.6	1.9	5.8	13.6	42.6

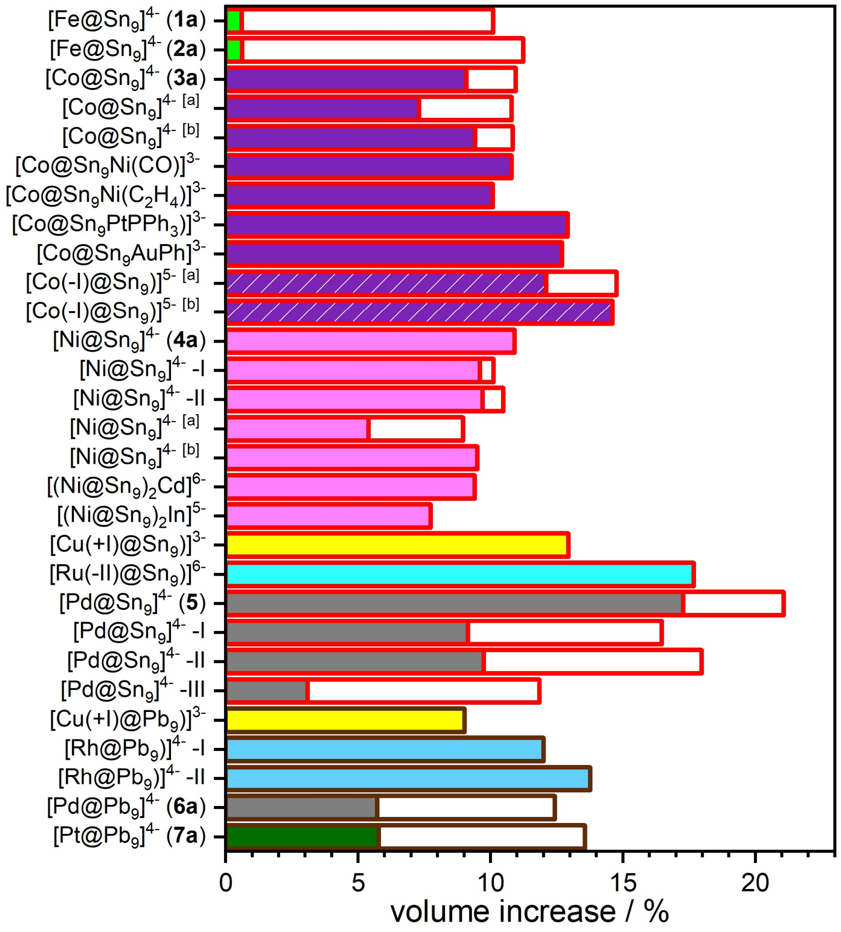

Figure S17: Volume increase of the endohedral clusters **1a** to **7a** and a comparison to the literature known endohedral clusters in a bar chart. The coloured bar represents the volume increase of the endohedral clusters in comparison to the empty ones, for partially occupied *TM* atoms positions the normalized volume, i.e. the volume increase extrapolated to a *TM* occupation of 100 %, is shown as an empty bar. Therefore, the sum of both bars represents the normalized volume increase. The bar filling color represents the central TM atom, Fe, Co, Ni, Cu, Ru, Pd, Rh, Pt, Sn and Pb are shown as bright green, purple, pink, yellow, bright blue, grey, blue, dark green, respectively. The bar frame colors indicate the cluster framework element, red and brown for Sn and Pb, respectively. For clarity, the bars for Sn_9 filled with negatively charged Co(−I) are dashed with white lines. If a cluster was present in more than one structure, it is indicated by [a] and [b]. In compounds with two crystallographically independent cluster anions, the anions are individually analyzed and displayed with roman numbers. The second bar has the same frame color as the first one and the bar filling is always empty. References see Table S9.

6. Crystallographic details

Table S10: Crystallographic data and details of the structure determinations of compounds **1** and **2.**

compound	$[Li_4(en)_8][Fe_{0.059(3)}@Sn_9]$ (**1**)	$[K([18]crown\text{-}6)]_2K_2[Fe_{0.055(4)}@Sn_9]\cdot$ 1.5 en (**2**)
formula	$C_{16}H_{64}Fe_{0.06}Li_4N_{16}Sn_9$	$C_{27}H_{60}Fe_{0.06}K_4N_3O_{12}Sn_9$
fw [g·mol^{-1}]	1580.15	1846.46
space group	$P\bar{1}$ (no. 2)	$P2_1/n$ (no. 14)
a [Å]	12.2812(4)	10.4131(2)
b [Å]	12.5497(4)	25.7429(7)
c [Å]	17.7177(5)	20.7178(4)
α [°]	75.249(2)	90
β [°]	71.320(2)	102.491(2)
γ [°]	62.721(2)	90
V [Å^3]	2280.27(13)	5422.2(2)
Z	2	4
T [K]	150(2)	150(2)
λ [Å]	0.71073	0.71073
ρ_{calcd} [g·cm^{-3}]	2.301	2.262
μ [mm^{-1}]	4.889	4.439
collected reflections	49827	82581
R_{int}	0.0259	0.0758
independent reflections	8945	10639
reflections > 2 $\sigma(I)$	7236	8383
parameters / restraints	411 / 0	526 / 12
R_1 [I > 2 $\sigma(I)$ / all data]	0.0273 / 0.0383	0.0368 / 0.0529
wR_2 [I > 2 $\sigma(I)$ / all data]	0.0694 / 0.0721	0.0825 / 0.0893
goodness of fit	1.013	1.028
max./min. difference electron density [e/Å^{-3}]	1.744 / −1.305	1.663 / −1.250
depository no.	CCDC-2300165	CCDC-2300166

Table S11: Crystallographic data and details of the structure determinations of compounds **3**, **4** and **5**.

compound	$[Li_4(en)_8][Co_{0.827(5)}@Sn_9]$ (**3**)	$[Li_4(en)_8][Ni@Sn_9]$ (**4**)	$[Li_4(en)_8][Pd_{0.824(4)}@Sn_9]$ (**5**)
formula	$C_{16}H_{64}Co_{0.83}Li_4N_{16}Sn_9$	$C_{16}H_{64}Li_4N_{16}NiSn_9$	$C_{16}H_{64}Pd_{0.82}Li_4N_{16}Sn_9$
fw [g·mol^{-1}]	1625.56	1635.51	1664.58
space group	*Pbcn* (no. 60)	*Pbcn* (no. 60)	*Pbcn* (no. 60)
a [Å]	14.2610(4)	14.3235(4)	14.3052(7)
b [Å]	17.3545(4)	17.3470(4)	17.3899(7)
c [Å]	18.8690(4)	18.8930(5)	18.9589(11)
α [°]	90	90	90
β [°]	90	90	90
γ [°]	90	90	90
V [Å^3]	4669.9(2)	4694.3(2)	4716.3(4)
Z	4	4	4
T [K]	150(2)	150(2)	150(2)
λ [Å]	0.71073	0.71073	0.71073
ρ_{calcd} [g·cm^{-3}]	2.312	2.314	2.344
μ [mm^{-1}]	5.041	5.122	5.015
collected reflections	50558	41393	29654
R_{int}	0.0403	0.0279	0.1054
independent reflections	4590	4621	4648
reflections > 2 σ(*I*)	3817	4064	3037
parameters / restraints	210 / 0	209 / 0	210 / 0
R_1 [*I* > 2 σ(*I*) / all data]	0.0347 / 0.0460	0.0216 / 0.0277	0.0526 / 0.0994
wR_2 [*I* > 2 σ(*I*) / all data]	0.0714 / 0.0755	0.0534 / 0.0554	0.1093 / 0.1288
goodness of fit	1.036	1.038	1.019
max./min. difference electron density [e/Å^{-3}]	2.131 / −2.956	0.618 / −0.731	1.682 / −1.367
depository no.	CCDC-2300167	CCDC-2300168	CCDC-2300170

Table S12: Crystallographic data and details of the structure determinations of compounds **6**, and **7**.

compound	$[Li_4(en)_8][Pd_{0.460(5)}@Pb_9]$ (**6**)	$[Li_4(en)_8][Pt_{0.426(4)}@Pb_9]$ (**7**)
formula	$C_{16}H_{64}Li_4N_{16}Pb_9Pd_{0.46}$	$C_{16}H_{64}Li_4N_{16}Pb_9Pt_{0.43}$
fw [g·mol^{-1}]	2422.24	2456.70
space group	*Pbcn* (no. 60)	*Pbcn* (no. 60)
a [Å]	14.2941(6)	14.2677(3)
b [Å]	17.4447(8)	17.4540(4)
c [Å]	19.0306(11)	18.9631(5)
α [°]	90	90
β [°]	90	90
γ [°]	90	90
V [Å^3]	4745.4(4)	4722.35(19)
Z	4	4
T [K]	150(2)	150(2)
λ [Å]	0.71073	0.71073
ρ_{calcd} [g·cm^{-3}]	3.390	3.455
μ [mm^{-1}]	32.002	33.248
collected reflections	28704	47963
R_{int}	0.0830	0.0990*
independent reflections	4668	4644
reflections > 2 $\sigma(I)$	3367	3352
parameters / restraints	210 / 0	210 / 0
R_1 [I > 2 $\sigma(I)$ / all data]	0.0410 / 0.0741	0.0547 / 0.0916
wR_2 [I > 2 $\sigma(I)$ / all data]	0.0787 / 0.0890	0.0915 / 0.1059
goodness of fit	1.024	1.134
max./min. diff. el. density [e/Å^{-3}]	2.151 / −2.817	2.107 / −2.354
depository no.	CCDC-2300169	CCDC-2300171

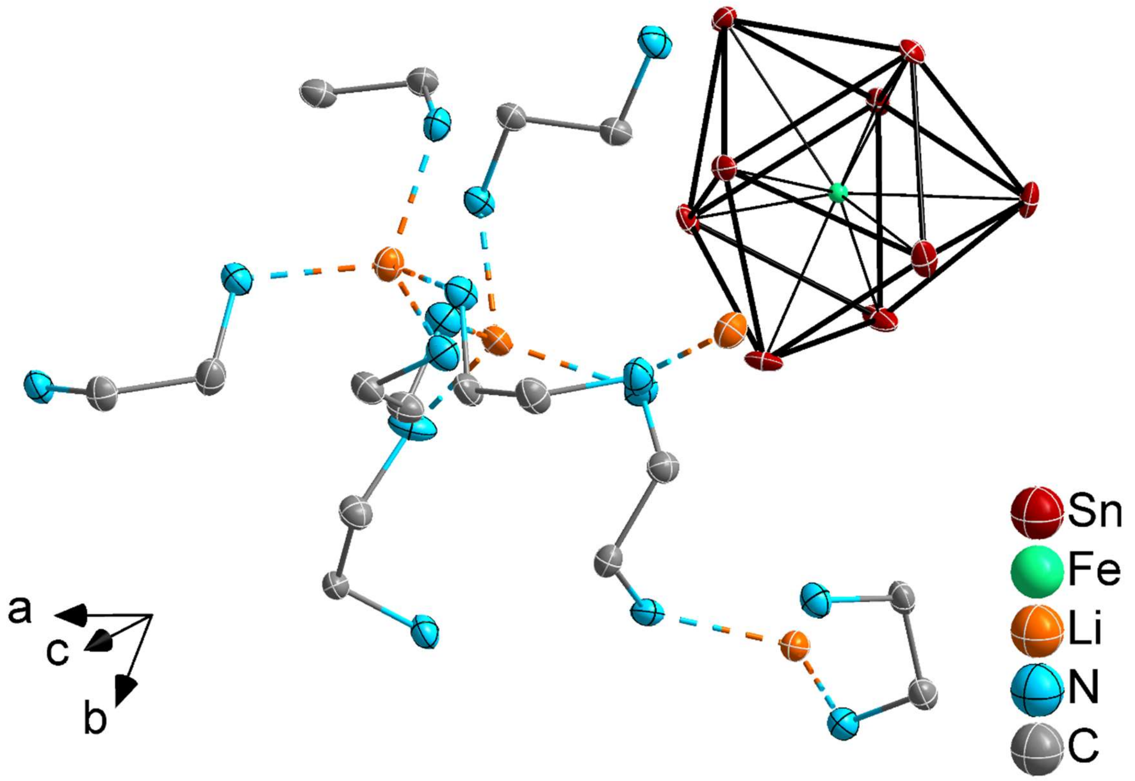

Figure S18: The asymmetric unit of compound **1**. Thermal ellipsoids are drawn at 50% probability. Hydrogen atoms are omitted for clarity.

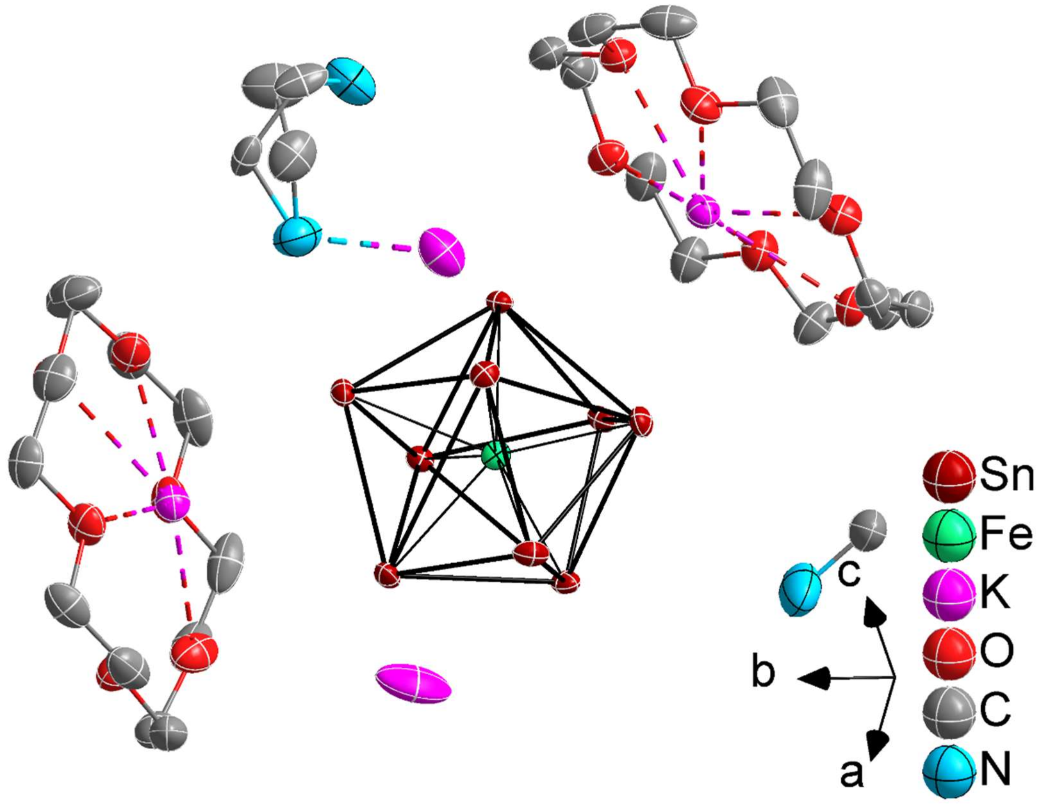

Figure S19: The asymmetric unit of compound **2**. Thermal ellipsoids are drawn at 50% probability. Hydrogen atoms are omitted for clarity.

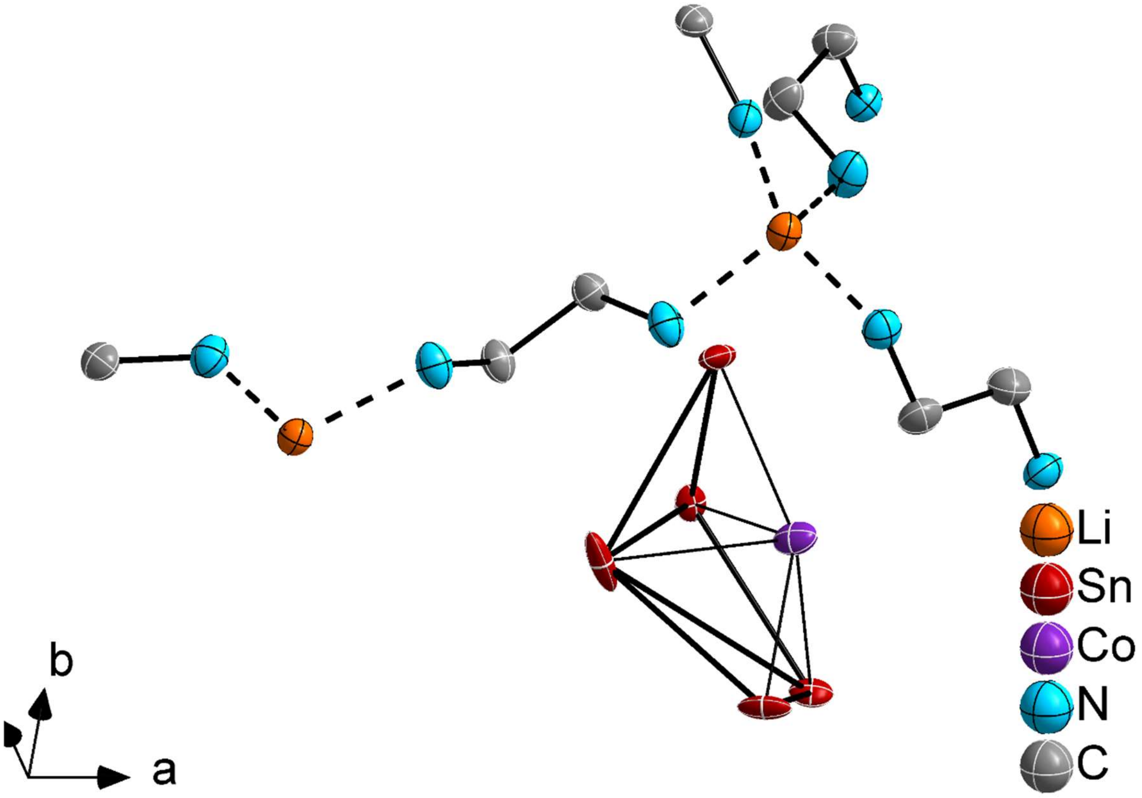

Figure S20: The asymmetric unit of compound **3**. Thermal ellipsoids are drawn at 50% probability. Hydrogen atoms are omitted for clarity.

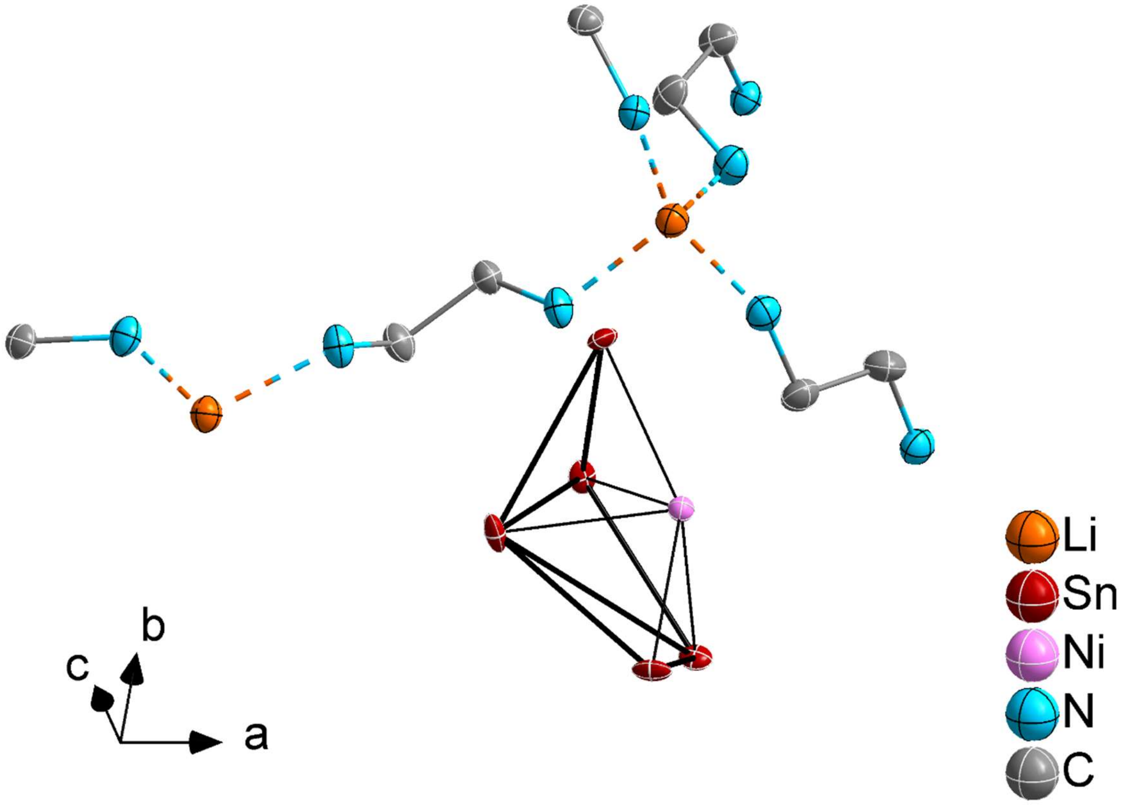

Figure S21: The asymmetric unit of compound **4**. Thermal ellipsoids are drawn at 50% probability. Hydrogen atoms are omitted for clarity.

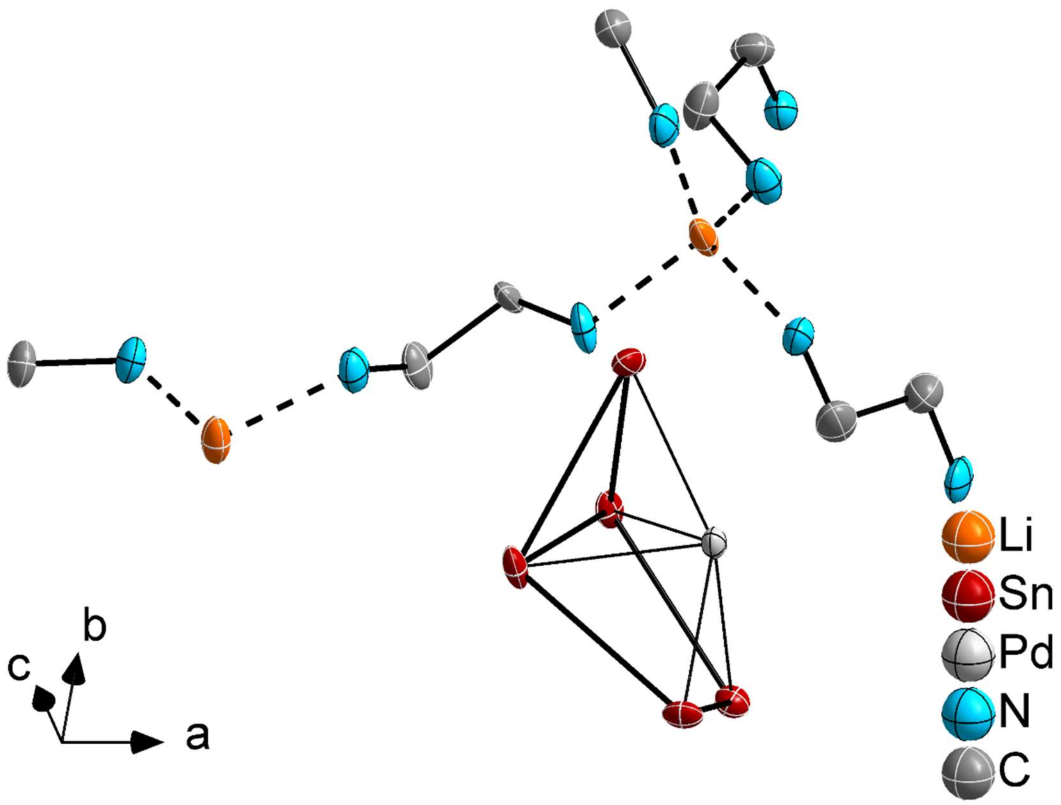

Figure S22: The asymmetric unit of compound **5**. Thermal ellipsoids are drawn at 50% probability. Hydrogen atoms are omitted for clarity.

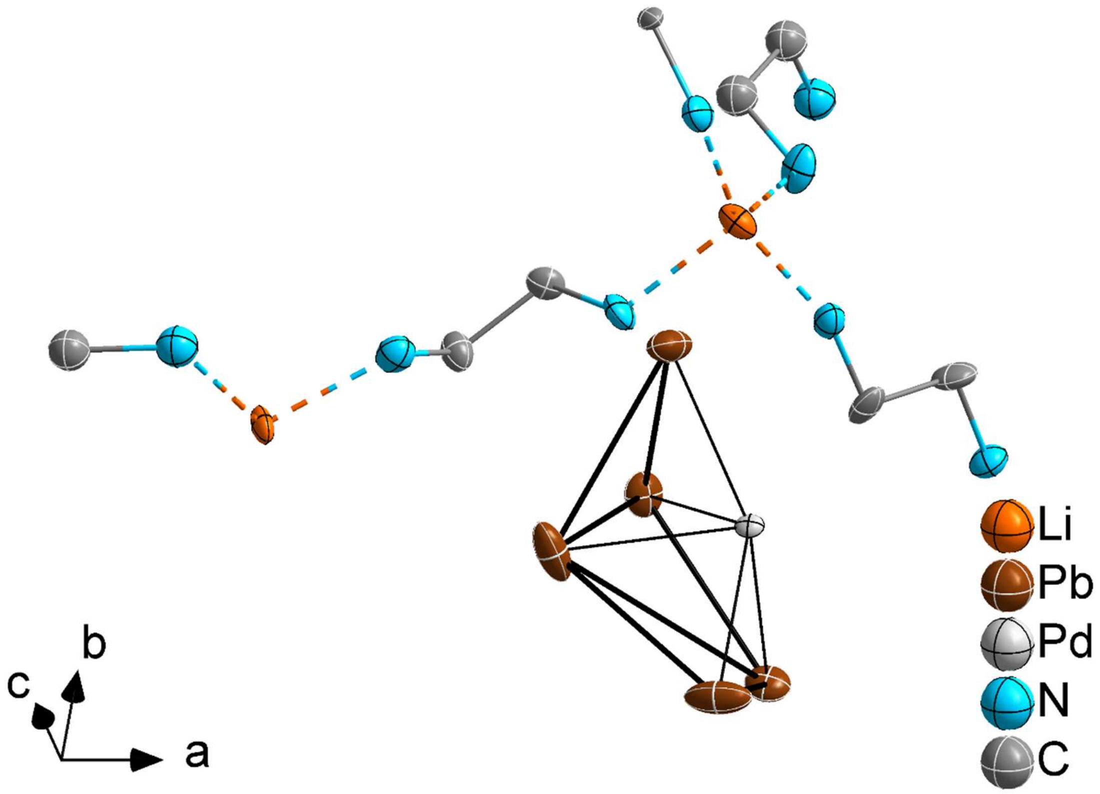

Figure S23: The asymmetric unit of compound **6**. Thermal ellipsoids are drawn at 50% probability. Hydrogen atoms are omitted for clarity.

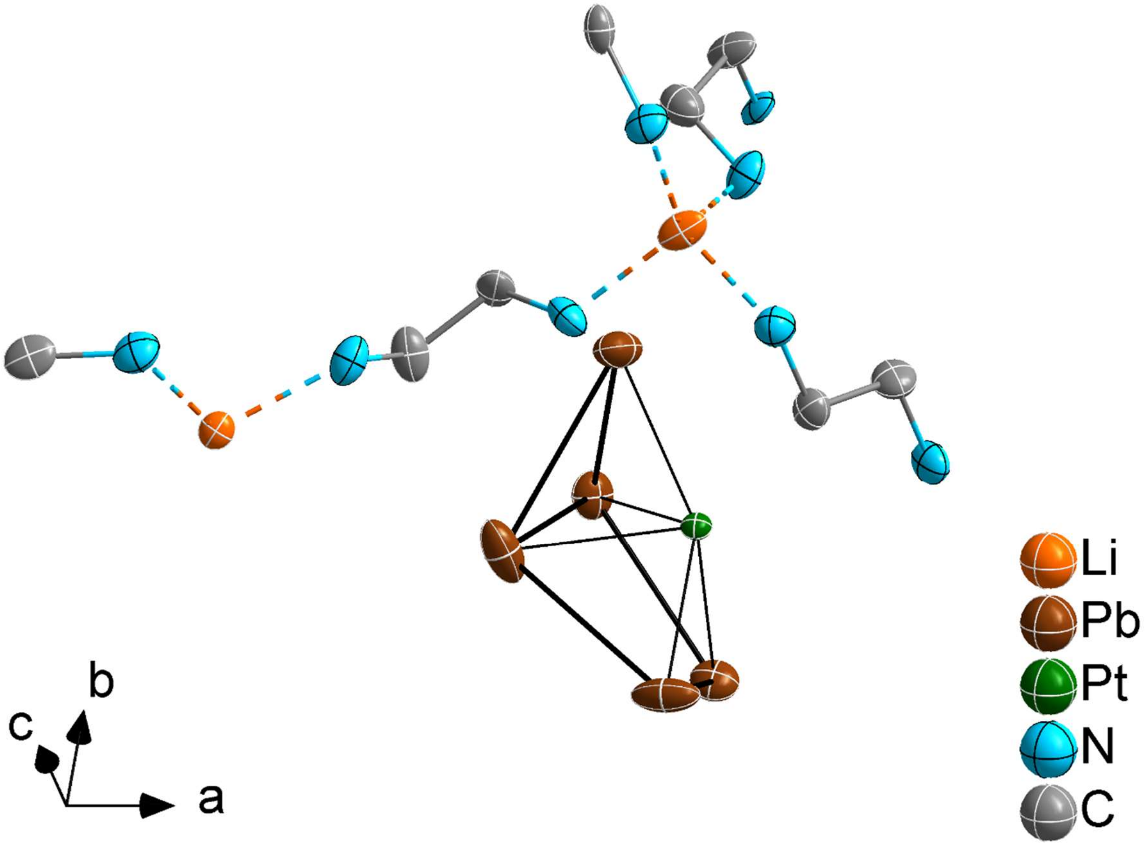

Figure S24: The asymmetric unit of compound **7**. Thermal ellipsoids are drawn at 50% probability. Hydrogen atoms are omitted for clarity.

Table S13. Selected interatomic distances in **1**.

Atom 1	Atom 2	distance [Å]	Atom 1	Atom 2	distance [Å]
Sn1	Sn2	2.9564(5)	Li1	N1	2.047(11)
	Sn4	2.9641(8)		N6	2.074(9)
	Sn5	2.9362(5)		N16	2.078(9)
	Sn6	3.0070(4)		N4	2.094(10)
Sn2	Sn3	2.9675(8)	Li2	N2	2.062(9)
	Sn6	2.9646(5)		N3	2.064(10)
	Sn7	2.9947(5)		N7	2.083(8)
Sn3	Sn4	2.9784(5)		N5	2.084(11)
	Sn7	2.9653(5)	Li3	N9	2.068(8)
	Sn8	2.9359(5)		N11	2.070(12)
Sn4	Sn5	2.9589(5)		N8	2.075(7)
	Sn8	2.9838(6)		N13	2.090(10)
Sn5	Sn9	2.9683(5)	Li4	N10	2.063(10)
	Sn6	3.2880(5)		N15	2.075(7)
Sn6	Sn7	3.0907(7)		N14	2.085(9)
	Sn9	2.9554(6)		N12	2.086(9)
Sn7	Sn9	2.9293(4)			
Sn8	Sn9	2.9796(6)			
Fe	Sn1	2.626(12)			
	Sn2	2.551(9)			
	Sn3	2.585(11)			
	Sn4	2.559(9)			
	Sn5	2.416(8)			
	Sn6	2.479(12)			
	Sn7	2.469(9)			
	Sn8	2.457(13)			
	Sn9	2.841(8)			

Table S14. Selected interatomic distances in **2**

Atom 1	Atom 2	distance [Å]	Atom 1	Atom 2	distance [Å]
Sn1	Sn2	2.9467(6)	K1	O1	2.896(4)
	Sn4	2.9289(7)		O2	2.823(4)
	Sn5	2.9920(7)		O3	2.890(4)
	Sn6	2.9879(6)		O4	2.941(4)
Sn2	Sn3	2.9800(7)		O5	2.813(5)
	Sn6	2.9890(7)		O6	2.906(4)
	Sn7	2.9616(6)	K2	O7	2.972(5)
Sn3	Sn4	2.9563(6)		O8	2.831(4)
	Sn7	2.9625(6)		O9	2.925(5)
	Sn8	2.9874(7)		O10	2.904(5)
Sn4	Sn5	2.9964(7)		O11	2.952(4)
	Sn8	2.9401(6)		O12	2.861(5)
Sn5	Sn6	3.1735(6)	K3	N1	3.072(7)
	Sn8	3.2187(6)		N2	2.761(8)
	Sn9	2.9597(5)			
Sn6	Sn7	3.2259(6)			
	Sn9	2.9674(7)			
Sn7	Sn8	3.2702(6)			
	Sn9	2.9421(7)			
Sn8	Sn9	2.9498(6)			
Fe	Sn1	2.571(11)			
	Sn2	2.635(13)			
	Sn3	2.564(13)			
	Sn4	2.575(12)			
	Sn5	2.456(12)			
	Sn6	2.478(13)			
	Sn7	2.462(11)			
	Sn8	2.450(13)			
	Sn9	2.812(13)			

Table S15. Selected interatomic distances in **3**

Atom 1	Atom 2	distance [Å]	Atom 1	Atom 2	distance [Å]
Sn1	Sn2	2.9942(5)	Li1	N1	2.040(11)
	Sn4	3.0310(6)		N3	2.063(11)
Sn2	Sn3	3.0309(6)		N5	2.068(11)
	Sn4	3.0581(7)		N7	2.089(10)
	Sn5	3.1177(7)	Li2	N2	2.047(10)
Sn3	Sn4	3.0121(7)		N4	2.103(10)
	Sn5	2.9818(7)		N6	2.064(10)
Sn3'	Sn5	3.0052(6)		N8	2.043(11)
Sn4	Sn5	3.1021(7)			
Co	Sn1	2.6264(13)			
	Sn2	2.5723(6)			
	Sn3	2.7005(7)			
	Sn4	2.5936(6)			
	Sn5	2.5835(10)			

Table S16. Selected interatomic distances in **4.**

Atom 1	Atom 2	distance [Å]	Atom 1	Atom 2	distance [Å]
Sn1	Sn2	3.0030(4)	Li1	N1	2.050(6)
	Sn4	3.0445(4)		N3	2.058(6)
Sn2	Sn3	3.0352(5)		N5	2.057(6)
	Sn4	3.0935(5)		N7	2.106(6)
	Sn5	3.0968(5)	Li2	N2	2.059(6)
Sn3	Sn4	3.0203(5)		N4	2.097(6)
	Sn5	3.0113(5)		N6	2.070(6)
Sn3'	Sn5	3.0391(4)		N8	2.054(6)
Sn4	Sn5	3.0893(5)			
Ni	Sn1	2.6484(6)			
	Sn2	2.5963(4)			
	Sn3	2.6797(4)			
	Sn4	2.6288(4)			
	Sn5	2.5954(5)			

Table S17. Selected interatomic distances in **5**.

Atom 1	Atom 2	distance [Å]	Atom 1	Atom 2	distance [Å]
Sn1	Sn2	3.0578(10)	Li1	N1	2.051(17)
	Sn4	3.1034(10)		N3	2.052(19)
Sn2	Sn3	3.0936(11)		N5	2.074(17)
	Sn4	3.1179(11)		N7	2.10(2)
	Sn5	3.1321(11)	Li2	N2	2.044(18)
Sn3	Sn5	3.0654(12)		N4	2.093(18)
	Sn4	3.0756(12)		N6	2.062(18)
Sn3'	Sn5	3.0952(10)		N8	2.03(2)
Sn4	Sn5	3.1133(11)			
Pd	Sn1	2.6880(15)			
	Sn2	2.6460(9)			
	Sn3	2.7191(9)			
	Sn4	2.6747(8)			
	Sn5	2.6398(11)			

Table S18. Selected interatomic distances in **6** (left) and in **7** (right).

**Atom 1**	**Atom 2**	**distance [Å]**	**Atom 1**	**Atom 2**	**distance [Å]**
Pb1	Pb2	3.1091(7)	Pb1	Pb2	3.1088(10)
	Pb4	3.1551(7)		Pb4	3.1637(11)
Pb2	Pb3	3.1520(7)	Pb2	Pb3	3.1528(10)
	Pb4	3.1825(8)		Pb4	3.1801(13)
	Pb5	3.2285(8)		Pb5	3.2339(10)
Pb3	Pb4	3.1377(9)	Pb3	Pb4	3.1335(12)
	Pb5	3.1237(8)		Pb5	3.1190(12)
	Pb5‘	3.1416(7)		Pb5‘	3.1459(11)
Pb4	Pb5	3.2169(8)	Pb4	Pb5	3.2177(12)
Pd	Pb1	2.741(2)	Pt	Pb1	2.751(2)
	Pb2	2.6837(10)		Pb2	2.6796(10)
	Pb3	2.8172(12)		Pb3	2.8156(12)
	Pb4	2.7138(10)		Pb4	2.7153(11)
	Pb5	2.6594(17)		Pb5	2.6609(16)
Li1	N1	2.08(2)	Li1	N1	2.07(3)
	N3	2.04(2)		N3	2.12(3)
	N5	2.10(2)		N5	2.08(3)
	N7	2.09(2)		N7	2.03(3)
Li2	N2	2.04(2)	Li2	N2	2.06(3)
	N4	2.07(2)		N4	2.06(3)
	N6	2.06(2)		N6	2.08(3)
	N8	2.09(2)		N8	2.05(3)

7. Cartesian coordinates of optimized structures

Table S19. Cartesian coordinates of the optimised structure of $[Co@Sn_9]^{4-}$.

Atom	x	y	z
Co	14.12596979767554	14.57316142632465	14.06202586672079
Sn	15.12184442388168	12.55808908545825	12.71263390078635
Sn	12.22796155323993	13.33816529266712	12.84135982873356
Sn	14.70636116114863	15.47439464137410	16.45431056974489
Sn	14.08614280874879	17.14208796212468	14.03929614868967
Sn	16.54241358442958	15.48288770335133	13.61166846241965
Sn	13.30460604726878	12.56207664423453	15.53227028518493
Sn	16.18743595299994	13.15816411581721	15.39156078256227
Sn	14.34483285447173	15.23207819102040	11.59128645911300
Sn	11.96243181613515	15.23468493762748	15.28108769604462

Table S20. Cartesian coordinates of the optimised structure of $[Fe@Sn_9]^{4-}$.

Atom	x	y	z
Fe	14.20505679608567	14.52247044879041	14.11225086424997
Sn	15.14644434832207	12.51160710679029	12.68347432905173
Sn	12.16305814285005	13.37138448640103	12.79877394948313
Sn	14.72006014928080	15.48889960525713	16.51198444600973
Sn	14.02390744389945	17.19155916648451	13.99831680572657
Sn	16.60056925510808	15.49662607431882	13.60184905676362
Sn	13.28890641150833	12.51662758158586	15.56739999947981
Sn	16.28629292322376	13.09300248093373	15.45528589236898
Sn	14.28180541213031	15.28124645157562	11.54369160307026
Sn	11.89389911759116	15.28236659786230	15.24447305379586

7. References

[1] V. Queneau, S. C. Sevov, *Inorg. Chem.* 1998, ***37***, 1358-1360.

[2] C. Hoch, Wendorff, M. & Röhr, C., *Acta Cryst.* 2002, ***C59***, i45-i46.

[3] G. Sheldrick, *Acta Crystallogr. Sect. A* 2008, ***64***, 112-122.

[4] G. Sheldrick, *Acta Crystallogr. Sect. C* 2015, ***71***, 3-8.

[5] S. Stoll, A. Schweiger, *J. Magn. Reson.* 2006, ***178***, 42-55.

[6] L. Link, R. Niewa, *J. Appl. Crystallogr.* 2023, ***56***.

[7] F. Neese, F. Wennmohs, U. Becker, C. Riplinger, *J. Phys. Chem.* 2020, ***152***.

[8] F. Neese, *Wiley Interdiscip. Rev. Comput. Mol. Sci.* 2018, ***8***, e1327.

[9] R. A. Kendall, H. A. Früchtl, *Theor. Chem. Acc.* 1997, ***97***, 158-163.

[10] F. Neese, F. Wennmohs, A. Hansen, U. Becker, *Chem. Phys.* 2009, ***356***, 98-109.

[11] S. Grimme, *J. Phys. Chem.* 2005, ***109***, 3067-3077.

[12] N. B. Balabanov, K. A. Peterson, *J. Phys. Chem.* 2006, ***125***.

[13] J. G. Hill, K. A. Peterson, *J. Phys. Chem.* 2014, ***141***.

[14] StoeWinXPow, STOE, Germany, Darmstadt, **2003**.

[15] S. Scharfe, T. F. Fässler, S. Stegmaier, S. D. Hoffmann, K. Ruhland, *Chem. Eur. J.* 2008, ***14***, 4479-4483.

[16] V. Hlukhyy, H. He, L.-A. Jantke, T. F. Fässler, *Chem. Eur. J.* 2012, ***18***, 12000-12007.

[17] V. Hlukhyy, S. Stegmaier, L. van Wüllen, T. F. Fässler, *Chem. Eur. J.* 2014, ***20***, 12157-12164.

[18] C. Liu, L.-J. Li, X. Jin, J. E. McGrady, Z.-M. Sun, *Inorg. Chem.* 2018, ***57***, 3025-3034.

[19] B. J. L. Witzel, W. Klein, J. V. Dums, M. Boyko, T. F. Fässler, *Angew. Chem. Int. Ed.* 2019, ***58***, 12908-12913.

[20] M. M. Gillett-Kunnath, J. I. Paik, S. M. Jensen, J. D. Taylor, S. C. Sevov, *Inorg. Chem.* 2011, ***50***, 11695-11701.

[21] Y.-N. Yang, L. Qiao, Z.-M. Sun, *Chin. Chem. Lett.* 2023, ***34***, 107207.
